# A follicular regulatory Innate Lymphoid Cell population impairs interactions between germinal center Tfh and B cells

**DOI:** 10.1038/s42003-021-02079-0

**Published:** 2021-05-12

**Authors:** Margaret H. O’Connor, Roshell Muir, Marita Chakhtoura, Michael Fang, Eirini Moysi, Susan Moir, Alison J. Carey, Alyssa Terk, Carmen N. Nichols, Talibah Metcalf, Constantinos Petrovas, Mark J. Cameron, Virginie Tardif, Elias K. Haddad

**Affiliations:** 1 The Department of Medicine, Division of Infectious Diseases & HIV Medicine, Drexel University College of Medicine, Philadelphia, PA USA; 2 The Department of Molecular and Cellular Biology and Genetics, Drexel University College of Medicine, Philadelphia, PA USA; 3 The Department of Population and Quantitative Health Services, Case Western Reserve University, Cleveland, OH USA; 4 Tissue Analysis Core, Vaccine Research Center, National Institute of Allergy and Infectious Diseases, National Institutes of Health, Bethesda, MD USA; 5 The Laboratory of Immunoregulation, National Institute of Allergy and Infectious Diseases, National Institutes of Health, Bethesda, MD USA; 6 The Department of Microbiology and Immunology, Drexel University College of Medicine, Philadelphia, PA USA; 7 Department of Pediatrics, St. Christopher’s Hospital for Children, Drexel University College of Medicine, Philadelphia, PA USA; 8 The Department of Otolaryngology, St. Christopher’s Hospital for Children, Drexel University College of Medicine, Philadelphia, PA USA; 9 Normandy University, UniRouen, INSERM, UMR1096 (EnVI Laboratory), Rouen, France

**Keywords:** Lymphocyte activation, Germinal centres, Innate lymphoid cells

## Abstract

Innate Lymphoid Cells (ILCs) are immune cells typically found on mucosal surfaces and in secondary lymphoid organs where they regulate the immune response to pathogens. Despite their key role in the immune response, there are still fundamental gaps in our understanding of ILCs. Here we report a human ILC population present in the follicles of tonsils and lymph nodes termed follicular regulatory ILCs (ILC_FR_) that to our knowledge has not been previously identified. ILC_FR_ have a distinct phenotype and transcriptional program when compared to other defined ILCs. Surprisingly, ILC_FR_ inhibit the ability of follicular helper T (Tfh) cells to provide B cell help. The localization of ILC_FR_ to the germinal centers suggests these cells may interfere with germinal center B cell (GC-B) and germinal center Tfh cell (GC-Tfh) interactions through the production of transforming growth factor beta (TGF-β. Intriguingly, under conditions of impaired GC-Tfh-GC-B cell interactions, such as human immunodeficiency virus (HIV) infection, the frequency of these cells is increased. Overall, we predict a role for ILC_FR_ in regulating GC-Tfh-GC-B cell interactions and propose they expand in chronic inflammatory conditions.

## Abstract (web summary)

Margaret O’Connor et al. report a new Innate Lymphoid Cell population in human tonsils and lymph nodes that inhibit the functional interaction of follicular helper T cells and germinal center B cells. They show that this cell population is expanded under chronic HIV infection and results in decreased antibody production, suggesting a potential role for these cells in diseases with dysregulated immune responses.

## Introduction

Innate lymphoid cells (ILCs) are located at many pathogen barriers including mucosal surfaces and secondary lymphoid organs (SLOs) where they can both sustain the integrity of the organ and promote lymphoid organogenesis itself^1^. ILCs are defined by the lack of a rearranged antigen receptor and other lineage surface markers and are subdivided into three groups: group 1 ILCs are similar to Type 1 helper T cells (Th1) and are characterized by their ability to secrete interferon gamma (IFN-γ) and respond to interleukin-12 (IL-12), IL-15, and IL-18; group 2 ILCs are analogous to Type 2 helper T cells (Th2) and produce the same cytokines including IL-5, IL-9, and IL-13 while responding to IL-25 and IL-33 stimulation; and group 3 ILCs which are similar to Type 17 and 22 helper T cells (Th17/22) and are able to produce IFN-γ, IL-17 and IL-22 after stimulation by IL-1β and IL-23^2^. A newly described ILC2 population (ILC2_10_) has recently been identified in mice that has regulatory properties due to their IL-10 production in response to IL-33 stimulation. ILC2_10_ also express the transcriptional regulatory factor ID3^3,4^. These cell types and the cytokines they secrete play an important role in regulating adaptive Th1, Th2, and type Th17/22 cell responses by promoting protective immunity and homeostasis^5,6^. Recent work has shown that circulating ILCs are irreversibly depleted in HIV infection and can be rescued upon viral control with antiretroviral (ART) therapy^7^, only if started during acute infection. These innate cells may be a crucial component leading to the adaptive CD4^+^ T cell immune cell dysfunction seen in late stages of HIV infection.

Germinal centers, found in SLOs, are critical for the success of humoral immunity and constitute a major target in the design of vaccines and immunotherapies. Specifically, germinal centers constitute the sites where long-lived memory B and plasma cells are generated^8–10^. They are also the sites where antibodies are generated, after B cells undergo affinity maturation and isotype switching^8–10^. This process is dependent on the interactions of a subset of CD4^+^ T cells called follicular helper T cells (Tfh) and GC-B cells. The GC-Tfh/GC-B cell interactions are tightly regulated by receptor/ligand interactions including CD40L/CD40 and ICOS/ICOSL as well as secretion of cytokines that can mediate GC-Tfh and GC-B cell help, such as IL-21, IL-4, IL-6, and IL-10^10–12^. All cells entering the B cell follicles (the dark zone) respond to CXCL13 gradients via upregulation of their CXCR5 receptor and modulation of CCR7 expression, which is required for their entry into the germinal center^13,14^. Within the germinal center, activated Tfh cells produce Interleukin-21 (IL-21), and express co-activation markers such as CD40L and ICOS, to allow for appropriate interaction and activation of the GC-B cells. Currently, there are fundamental gaps in understanding the cellular and molecular mechanisms during immune dysregulation and irregularities in germinal centers.

In this work, we describe a unique ILC population found in human tonsils and lymph nodes within the germinal center follicles. We find that they function in a regulatory manner, and as such, we name this newly identified cell population follicular regulatory ILCs (ILC_FR_), consistent with their location and function. These cells suppress the adaptive germinal center interaction between GC-Tfh and GC-B cells via production of TGF-β in vitro, resulting in a decreased production of IgG as well as germinal center-mediated helper cytokines. These cells are expanded in chronic viral infection and may play a role in the immune dysregulation observed in these chronic inflammatory states.

## Results

### A newly identified innate lymphoid cell exists in human tonsils

**Fig. 1:**
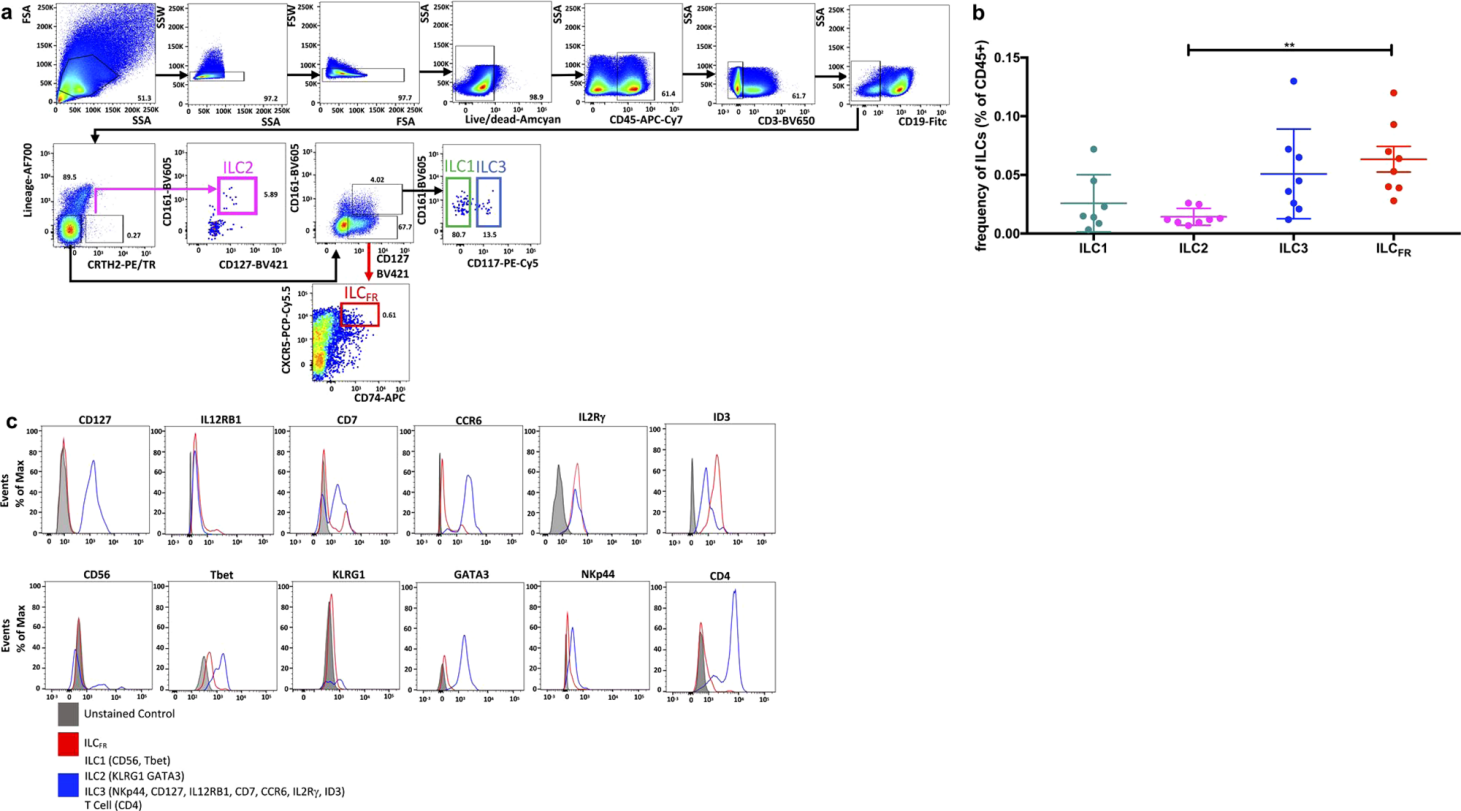
A unique ILC population exists in human tonsils. **a** Representative conventional flow cytometry plots from an adult tonsil showing the hierarchical phenotype gating strategy from singlet lymphocytes to the ILC1 (green), ILC2 (pink), and ILC3 (blue) and a unique ILC subset (ILC_FR_) (red) populations indicated by arrows and color-coded gates. (Lin = CD4, CD11b, CD11c, CD14, CD16). **b** Graph shows frequency of ILC1, ILC2, ILC3, and ILC_FR_ among CD45^+^ human tonsil mononuclear cells (*n* = 7 or 8 biologically independent tonsils per group, 3 independent experiments) (Ordinary one-way ANOVA with multiple comparisons; F = 5.2; **p = 0.0058; Mean ± SD). **c** Analysis of surface and transcription markers on ILC_FR_ by flow cytometry. Gray histograms depict fluorescence minus one (FMO) control of each antibody. Red lines indicate ILC_FR_. Blue lines denote ILC1 (for CD56, Tbet), ILC2 (KLRG1, GATA3), ILC3 (NKp44, ID3, CD127, ILR12RB1, CD7, CCR6, IL-2Rγ), and T cells (CD4) (*n* = 10 biologically independent tonsils per group, 3 independent experiments).

**Fig. 2:**
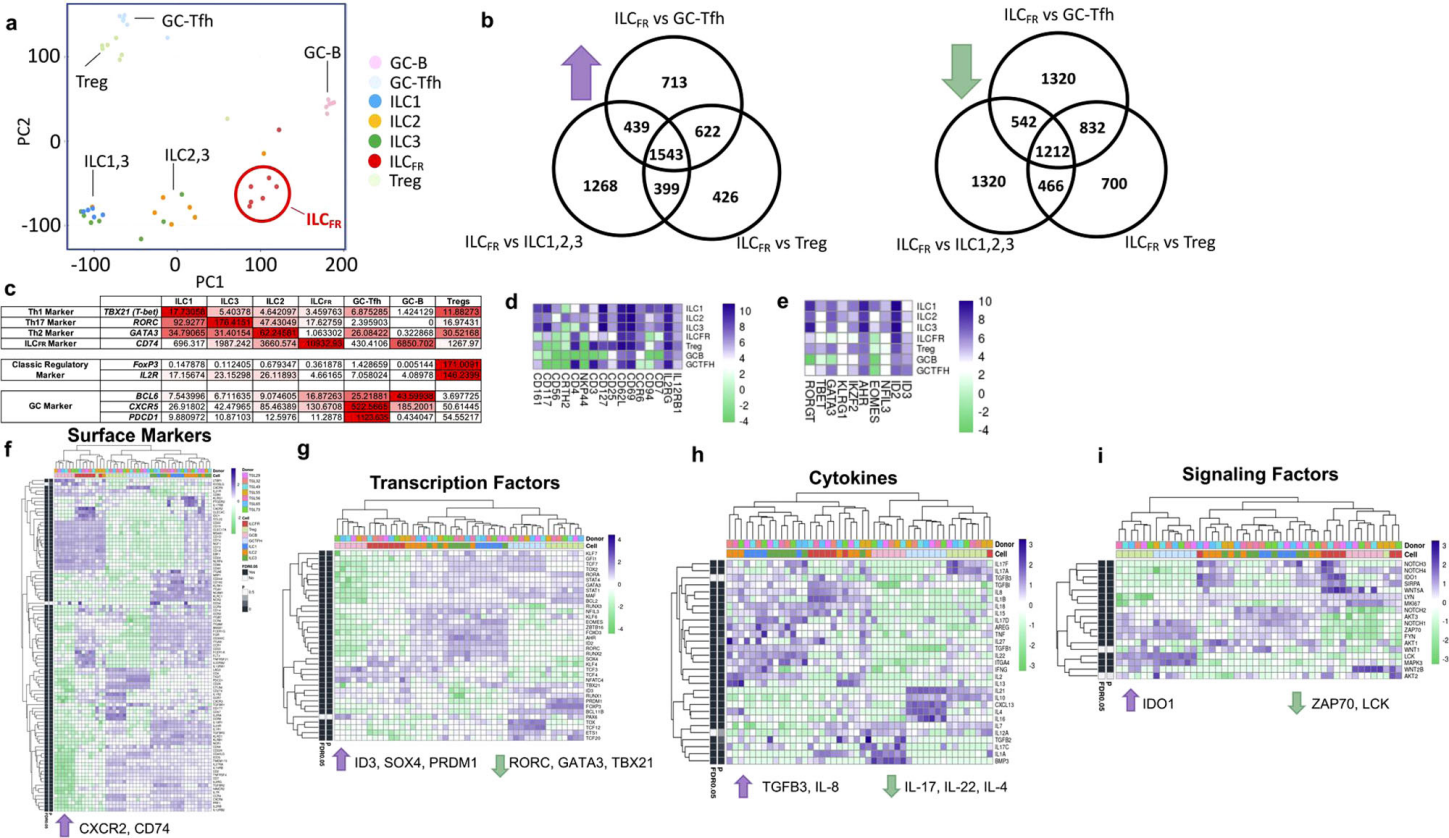
ILC_FR_ are distinct from other ILC subsets, Tregs, and GC-B or GC-Tfh cells. RNAseq analysis for GC-Tfh (light blue), GC-B (pink), Treg (light green), ILC1 (blue), ILC2 (orange), ILC3 (green), and ILC_FR_ (red) cells in healthy human tonsils. mRNA was extracted followed by gene expression profiling by using LIMMA in R Bioconductor software. **a** Principle component analysis of 40,000 unsupervised genes from the indicated cell populations. *n* = 7 biologically independent tonsils. **b** Venn diagram analysis of upregulated (left) or downregulated (right) differentially expressed genes comparing ILC_FR_ population to Treg, ILC1, 2 and 3, or GC-Tfh. *n* = 7 biologically independent tonsils. RPKM values were used to show major transcription factors from ILC subtypes, and important germinal center localization makers in **c** with a table demonstrating that ILC_FR_ are a distinct cell subtype and are localized to germinal center. **d** RNAseq analysis of ILC_FR_ for selected common ILC surface markers and **e** transcription factors. Genes related to innate and adaptive immunity were selected for heatmap analysis for **f** Surface markers, **g** Transcription Factors, **h** Cytokines, and **i** Signaling Factors. RNA of ILC_FR_ was extracted with RNeasy Micro Kit (Qiagen) and analyzed by NextSeq 550 (Illumina) sequencing on a High Output flow cell using a 75 base pairs, Paired End run. Human TMNCs *n* = 6 or 7 biologically independent tonsils averaged together for relative expression.

We examined by flow cytometry tonsillar mononuclear cells (TMNCs) from healthy individuals for the presence of ILCs and observed a unique population within this family of innate cells. These ILCs are phenotypically identifiable by examining a lineage negative^15^ population (CD11b^−^CD11c^−^CD14^−^CD16^−^CD4^−^) gated separately for Lin markers CD19^−^CD3^−^ that are also CD161^−^CD45^+^CD127^lo^CD74^+^CXCR5^+^ (Fig. 1a). These ILCs expressed similar amounts of common ILC markers when compared to ILC3 positive controls including IL-2Rγ, CD7, IL12RB1, and CCR6, but lacked or exhibited very low expression of markers for ILC1 (NKp44, CD56, Tbet), ILC2 (KLRG1, GATA3), and ILC3 (NKp44) or other leukocyte lineage markers^16–18^ shown by flow cytometry (Fig. 1c). ILC_FR_ lack the surface protein expression of IL7RA, a common surface marker for ILCs, which has recently been shown to have a redundant signaling role to IL15RA as ILCs persist in the small intestinal lamina propria (siLP) of adult and neonatal Il7ra KO mice. IL-15 sustains wild-type and Il7ra KO ILC survival in vitro and compensates for IL-7R deficiency^19^. This indicates that not all ILCs require expression of IL7RA, which is further evidenced by discovery of a CD127 low ILC population in humans^20^. Importantly, these ILCs simultaneously expressed unique markers including ID3 and CD74 (Fig. 1a, c). It has also been shown that ID3 and CD74 are expressed on a transcript level by murine intestinal regulatory ILCs^21^. Intriguingly, CD74 itself functions in a multifaceted role as part of the invariant chain of MHC II but also as the receptor for the inflammatory cytokine macrophage migration inhibitory factor (MIF). It has been shown that CD74 can act as a decoy receptor, which binds and neutralizes circulating MIF in the serum^22^. Hence, CD74 provides a potential newly identified mechanism for the negative regulation of MIF signal transduction and may be critically involved in controlling the pro-inflammatory actions of this cytokine. Another highly distinctive feature of ILC_FR_ is their similar expression levels of CXCR5 (Fig. 2c) when compared to GC-Tfh, gated as CD4^+^CD3^+^CD45RA^−^CXCR5^hi^PD1^hi^ (Supplementary Fig. 1a) and GC-B cells, gated as CD19^+^CD38^int^IgD^−^CD319^lo^ (Supplementary Fig. 1b). This similar expression level of CXCR5 RNA transcript suggests close physical proximity to other germinal center resident cells, and the ability to enter the germinal center follicles^23^. It is worth noting that ILC_FR_ lacked CD25, CD4, and FoxP3 expression (Figs. 1c, 2c) the distinguishing markers of T regulatory cells (CD4^+^CD3^+^CD45RA^−^CXCR5^−^PD1^−^CD25^+^CD127^lo^) (Supplementary Fig. 1c)^24^. ILC_FR_ also lacked classical ILC2/Th2 markers RORA, GATA3 (Figs. 1c, 2c, 2g), ICOS, IL2RA, KLRG1 (Figs. 1c, 2f) and do not express transcripts of known Th2/ILC2 cytokines IL-13, IL-5, or ILC2_10_ cytokine IL-10 (Fig. 2h). Additionally, the other three ILC subsets type 1, 2 and 3, were identified in TMNCs based on established gating strategies^7^ (Fig. 1a). Notably, ILC3 and ILC_FR_ subsets were found to be the two most dominant tissue resident ILC subpopulations with a statistically significant increase in frequency of ILC_FR_ over ILC2s. (Fig. 1b). All three other ILC subsets have varying expression of CXCR5 but markedly less Bcl6 expression suggesting location outside of the germinal center follicles themselves. Additionally, the other three subsets of ILCs also lack ID3 and CD74 expression allowing distinction from ILC_FR_ (Fig. 2c, g). Collectively, this new Lin^−^ CD161^−^CD45^+^CD127^lo^CD74^+^ID3^+^CXCR5^+^ cell population is present in human tonsils and displays distinct surface markers and transcription factors from ILC1s, ILC2s, ILC3s, and Tregs.

### ILC_FR_ are distinct from GC-B, GC-Tfh, Tregs, and other ILCs

In order to confirm that our newly identified ILC_FR_ represent a distinct subset that differ from other known ILCs, adaptive Tregs, GC-B or GC-Tfh populations, we performed RNA-sequencing analysis on sorted cells from human tonsils to characterize their transcriptional profiles. We isolated ILC_FR_ ex vivo from eight healthy human tonsils as defined by Lin^−^CD161^−^CD45^+^CD127^lo^CD74^+^CXCR5^+^. We also sorted six other cell subsets including ILC1s, ILC2s, ILC3s, GC-B cells, GC-Tfh cells, and Tregs as described in methods. Multidimensional scale (MDS) analysis of the seven subsets revealed that ILC_FR_ are transcriptionally distinct from other ILCs as well as from other T cell and B cell subsets (Fig. 2a). Our sorted population of ILC3s were comprised of additional specific subtypes (LTi or NCR^+^ ILC3s), hence the MDS analysis shows as expected, two clusters of ILC3s. One subset that is more similar to ILC1s, the NCR^+^ ILC3s, long established as transcriptionally similar to ILC1s with the ability to be reprogrammed into ILC1s with considerable cytotoxic capacity^25^. The other cluster consists of lymphoid tissue inducer like ILC3s, which are more distinct and groups closer to ILC2s^26^. ILC2s however, expressed unique transcriptional profiles distinct from ILC_FR_, ILC1 or NCR^+^ ILC3s. ILC_FR_ and other ILCs (ILC2 and ILC3s (LTi)) expressed multiple B cell associated, and antigen presenting cell associated transcripts (Supplementary Fig. 2c). This may be due to ability to present antigen, as ILC_FR_ do not express CD19 surface protein as proven by Flow cytometric analysis and RNA seq analysis (Figs. 1a, 2f). Overall, the MDS plot analysis confirmed the unique transcriptional profile of ILC_FR_ when compared to other ILCs, Tregs, GC-Tfh, and GC-B cells.

We additionally performed Venn diagram analysis to identify differentially expressed genes (DEGs) both upregulated (left) and downregulated (right) that are common or unique between (ILC_FR_) vs (ILC1,2,3), (ILC_FR_) vs (GC-Tfh), and (ILC_FR_) vs (Treg) (Fig. 2b). We found that ILC_FR_ have 426 upregulated unique DEGs with increased expression, such as *SMAD5* and *NOTCH2* compared to Tregs alone; and 700 genes that are downregulated such as *IL1R2*, *IL10RA*, *IL15RA*, *IL17A*, *CCR5*. When ILC_FR_ were compared to ILC1,2,3s, this newly identified ILC subset has 1268 upregulated genes, such as *IL21R*, *IL-21*, *IL10* and *ID3*; and 1320 downregulated genes including *EOMES*, *FASLG*, *TGFBR2*, *IL17RE*, *RORC*, and *NCAM1*. We also compared ILC_FR_ to Tregs and ILC1,2,3s simultaneously, which showed that ILC_FR_ have 399 upregulated genes over ILC1,2,3s and Tregs (ILC_FR_vsILC123 and ILC_FR_vsTregs) (Fig. 2b left) including genes such as *BCL6* and *TGFBI*, and 466 downregulated genes when compared to other ILCs or Tregs (Fig. 2b right), which include *IL23R*, *IL-22*, *TGFBR3*, *IL1R1*, and *IL2RA*. Moreover ILC_FR_ lack the expression of classical transcription factors necessary for the development of other ILC subsets and Tregs namely *RORC* (encoding for RORγt), important for ILC3 development, *TBX21* (encoding for Tbet) for ILC1 development^2,27^, *GATA3* for ILC2 subset development^28^, and *FOXP3* for Tregs^24^ (Fig. 2c, e), while sharing many common ILC genes including *IL2RG*, *CD7*, *CCR6*, and low expression of *IL-7R* (encoding for CD127) (Fig. 2d). Canonical Tregs were used for comparison in vitro and for sequencing instead of the recently described germinal center follicular regulatory T cells (Tfr) due to inconsistent suppressive functioning in our hands of Tfr in vitro. Additionally, they express unique identifying transcription factors such as *ID3* and *SOX4*, but also *ID2* and the common gamma chain (γc chain), which are required for ILC development^16,17^ (Fig. 2e–g). Of note was the increased expression of *SOX4* in ILC_FR_, which is upregulated via TGF-β signaling through SMAD3, and could be one mechanism by which ILC_FR_ are inhibiting the GC-Tfh and GC-B cell interaction. Furthermore, ILC_FR_ express intermediate levels of the transcription factor *NFIL3*, which plays a critical role in the development of mucosal tissue-associated innate lymphocytes^29^. Taken together, these results identified that the ILC_FR_ subset is transcriptionally unique with distinct gene expression profiles when compared to other ILC subsets, and adaptive regulatory cells in the human tonsil. We contend that these cells represent a newly identified innate lymphoid cell subset.

Examining cytokine receptor expression ex vivo, from sequencing human tonsillar ILC_FR_ indicated constitutive expression of *IL10RB*, *IL2RG*, and *IL21R*, suggesting these cells respond to IL-10, IL-2, and IL-21 signaling (Fig. 2f). On the other hand, cytokine transcriptome analysis of this cell population indicated intermediate levels of *TGFB2*, *TGFB3* and low *IL10* and *TGFB1* transcript expression profiles (Fig. 2h) suggesting that these cells might function though the TGF-β pathway. Intriguingly, there were high expression levels of *IL-8* and its receptor *CXCR2*, which are involved in cell migration towards immune sites^30^ (Fig. 2f, h). Similarly, ILC_FR_ expressed high gene transcripts of *CCR7* which is necessary, much like CXCR5, for recruitment into secondary lymphoid organs and germinal centers. CCR7 has been shown to be important for keeping the GC-Tfh and GC-B cells in close proximity to promote interactions that are required for efficient antibody responses^31^. Of additional interest, ILC_FR_ expressed high levels of *IDO1*, an intracellular enzyme, (Fig. 2i), which has been shown to lead to immune tolerance by the regulation of natural killer (NK) cells, T cells and Tregs^32^. The immune modulatory actions of IDO1 mainly result from starving the cellular microenvironment of tryptophan, and this reduction causes immunosuppressive effects^33^. Of note, ILC_FR_ showed upregulated transcript levels of many inflammatory cytokine surface receptors (CD74, IFNAR1 and 2, IFNGR1 and 2, IL12R, IL17R) suggesting that ILC_FR_ have the ability to sense inflammatory microenvironments and respond (Fig. 2f). RNA-sequencing-based pathway analysis further showed ILC_FR_ are immunosuppressive and unique compared to other ILCs (Supplementary Fig. 2a) and Tregs (Supplementary Fig. 2b). ILC_FR_ had decreased gene expression in the pathways for *CD40* signaling, *GATA3* signaling, *IL-2* and *IL-7* signaling, *TCR* signaling, and *41BB* signaling when compared to Treg and ILC1, 2, and 3; all important for immune cell activation, differentiation and survival (Supplementary Fig. 2a, 2b). These results tell us that human tonsillar ILC_FR_ express genes that suggest they have regulatory capacity.

### ILC_FR_ localize into the germinal center

**Fig. 3:**
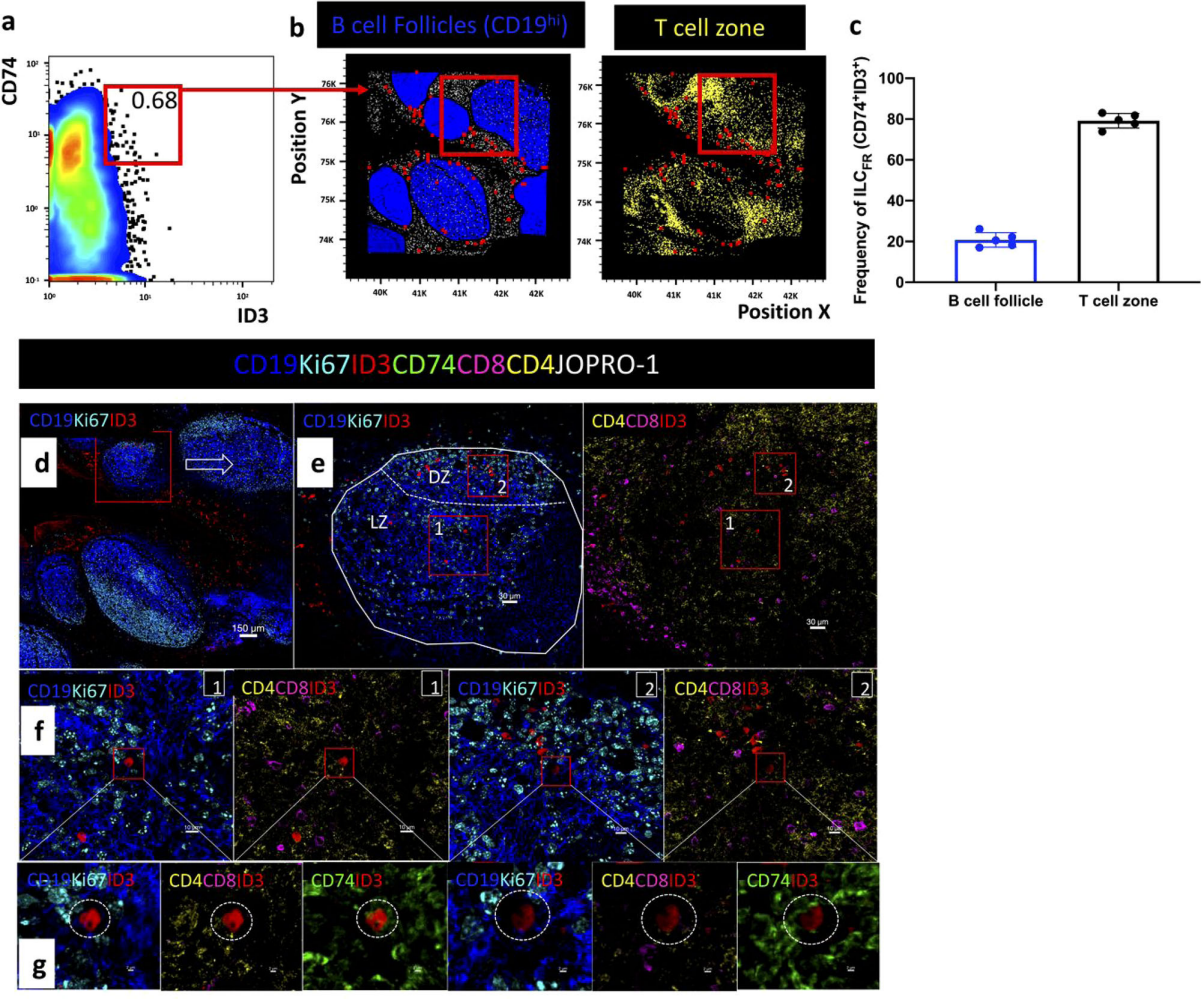
Distribution of CD74^+^ ID3^+^ ILC_FR_ in human tonsillar B cell follicles. **a** Histocytometry analysis showing the frequency of CD19− CD8− CD4− CD74+ ID3+ ILC_FR_ in a tonsillar tissue section (tonsil #1) and **b** Overlay showing the distribution of CD74+ ID3+ ILC_FR_ (red dots) with respect to CD19^+^ B cells (dark blue dots) and CD4^+^ T cells (yellow dots) in the same tonsil. **c** Bar graph summarizing the frequencies of intra- and extra- follicular ILC_FR_ (CD19− CD8− CD4−CD74+ ID3+) as a frequency of total ILC_FR_ in five tonsils. **d** Confocal images showing the tonsil area imaged, distribution of B cell follicles as denoted by CD19 (dark blue) and Ki67 (cyan) as well as ID3 positive cells (red). **e** Close up of a B cell follicle. Dotted lines demarcate the area of the follicle (LZ) as well as the dark zone (DZ) as defined by the density of Ki67 staining (cyan). B cells are shown in blue (CD19^+^), proliferating cells in cyan (Ki67^+^), ID3 in red, CD4 in yellow and CD8 in magenta. **f** Zoomed in details of the red rectangular enclosures shown in (**e**). The location of a CD74^+^ (green) ID3^+^ (red) cell is shown with respect to the positioning of CD19^+^ (blue) Ki67 (cyan) cells or CD8^+^ (magenta) and CD4^+^ (yellow) lymphoid cells. **g** Zoomed in close-ups confirming the positioning and phenotype of ILC_FR_ (CD19-CD4− CD8− CD74+ ID3+). Images were acquired at ×40 (NA 1.3) with no zoom. Images shown are sequential digital magnifications of 150 um (**d**), 30 um (**e**), 10 um (**f**), and 2 um (**g**). A total of five biologically independent human tonsils were imaged.

To demonstrate the localization of ILC_FR_ in situ within intact human tonsil tissue, we performed imaging analyses that allowed for the simultaneous detection of CD4, CD19, Ki67, CD8, JOPRO-1 (nucleus), CD74 and ID3 surface and intracellular markers for identification of ILC_FR_ within human tonsillar follicular areas. The images were further analyzed by histocytometry^34,35^. For visualization purposes, CD19^+^ B cells were stained in blue, Ki67 (proliferating cells) in cyan, ID3 in red, JOPRO-1 (cell nucleus marker) in gray, CD74 in green, CD4 (T cells) in yellow, and CD8 (T cells) in magenta to delineate separate cell populations at various magnifications. (Fig. 3d–g). B cell germinal center follicles were defined as areas displaying a high density of CD19 (blue). In line with previous reports, our imaging analysis showed that tonsils are highly populated with B cell follicles (blue (germinal centers)) and have T cell areas surrounding them (yellow)^36^ (Fig. 3b). Additionally, the CD19^−^CD4^−^CD8^−^ cells were analyzed in combination with the surface marker CD74 and transcription factor ID3, to specifically identify ILC_FR_ (red dots) which, as described above, are marked by the absence of CD8, CD4, and CD19 with positivity for ID3 in the nucleoplasm and CD74 on the cell surface (Fig. 3a–g, Supplementary Fig. 3a-b, 4a-d). We saw that these cells were able to be visualized within the human tonsils. Four additional human tonsils were analyzed, and imaging show similar ILC_FR_ localization trends (Supplementary Figs. 3, 4) revealing positioning that was either directly within the germinal center follicular CD19^+^ B cell areas themselves (in blue, Fig. 3a, b, Supplementary Fig. 3c) or in the T cell zone just outside the B cell follicles (yellow, Fig. 3b, Supplementary Fig. 4b). Isotype controls were also used to confirm ID3 staining (Supplementary Fig. 4e). While the majority of ILC_FR_ were located outside of the B cell follicle, around 22% were within the germinal center itself (Fig. 3c, Supplementary Fig. 3c-d). germinal centers are extremely dynamic microenvironments and this cytologic in situ snapshot suggests ILC_FR_ are able to modulate their receptor expression and exist both within and bordering germinal centers. This data highlights the uniqueness of ILC_FR_ in that they can indeed be located within the B cell area of germinal center follicles and are separate from B cells, CD4^+^ and CD8^+^ T cells.

### ILC_FR_ disrupt the GC-Tfh–GC-B cell interaction

**Fig. 4:**
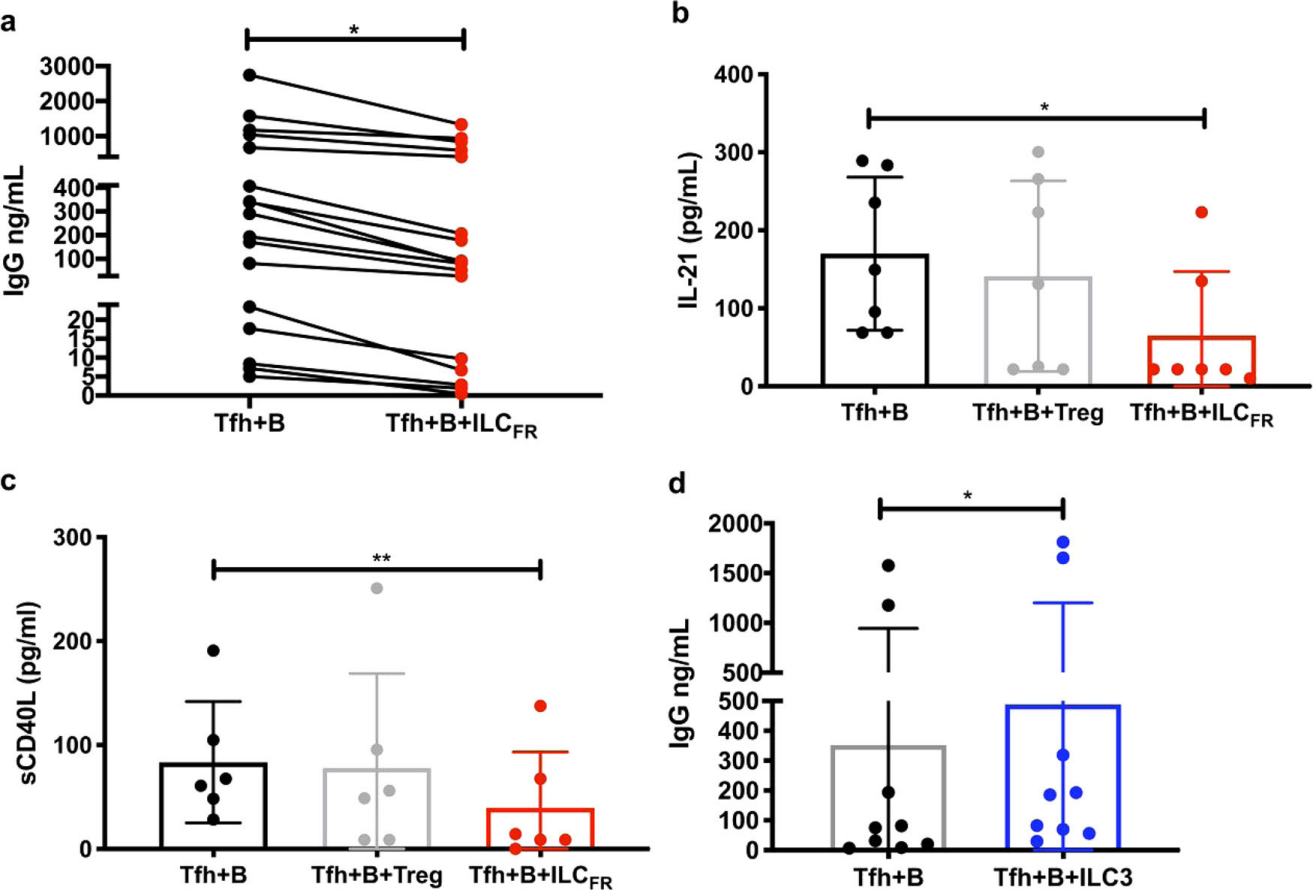
ILC_FR_ and ILC3s modify the GC-B and GC-Tfh interaction. **a** Production of IgG in the supernatants of 5-day co-culture of 30,000 GC-Tfh with 30,000 autologous GC-B cells plus or minus addition of 1000 ILC_FR_ in the presence of 100 ng/mL SEB superantigen measured by ELISA (*n* = 17 biologically independent tonsils per group, 5 independent experiments) (Paired, two-tailed parametric *t*-test; *p = 0.011; *t* = 2.899, df = 16, 95% CI (−432.2 to −67.08), Mean ± SD). **b** Production of IL-21 or **c** sCD40L analyzed by 28 plex Luminex. (*n* = 6–7 tonsils per group, 3 independent experiments) (Ordinary one-way ANOVA with multiple comparisons; IL-21: F = 3.542; *p < 0.0467; Mean ± SD), sCD40L: F = 6.081; **p < 0.0014; Mean ± SD). In **d** the graph shows IgG production in the supernatants of 5-day co-culture of 30,000 GC-Tfh with 30,000 autologous GC-B cells plus or minus addition of 3000 ILC3 in the presence of 100 ng/mL SEB superantigen measured by ELISA (*n* = 8 biologically independent tonsils per group, 4 independent experiments) (Paired, two-tailed parametric *t*-test; *p = 0.018; df = 7, *t* = 3.09, 95% CI: (9.47 to 71.15), Mean ± SD).

In an effort to decipher how ILC_FR_ have the potential to modulate GC-Tfh-GC-B interactions, we assessed their function using an in vitro co-culture assay^37,38^. Specifically, we determined whether ILC_FR_ could interfere with Tfh-dependent B cell responses. Tonsillar GC-Tfh cells (CD4^+^CD3^+^CD45RA^−^CXCR5^hi^PD1^hi^), defined previously^23,37^, were cultured with autologous GC-B cells (CD19^+^CD38^int^IgD^−^CD319^lo^)^37–39^ (Supplementary Fig. 1a, 1b) in the presence of the inflammatory super antigen staphylococcal enterotoxin B (SEB), as previously reported, which recapitulates an inflammatory reaction between the GC-Tfh and cognate GC-B cell^38,40^. These cells were cultured for five days with or without the addition of ILC_FR_ (Lin^−^CD45^+^CD127^lo^CD161^−^CD74^+^CXCR5^+^) (Supplementary Fig. 5) in a ratio of 30:30:1 (GC-Tfh: GC-B: ILC_FR_). The stringent nature of the gating strategy used to sort ILC_FR_ was included to emphasize ILC_FR_ are non-T, non-B, non-lineage (macrophage/DC/monocyte/NK/neutrophil) ILCs and to demonstrate the purity of the sorted ILC_FR_ cell population. With ILC_FR_ themselves being so rare in patients, purity was assessed by using extremely stringent gating and the reproducibility of sorting results (Supplementary Fig. 5). We have previously shown that five days of co-culture results in peak IgG production from TMNC^37,40^. The addition of low numbers of our ILC_FR_ (1000 cells) to co-cultures of GC-Tfh and GC-B cells significantly (*p* < 0.05) reduced the quantity of IgG produced by GC-B cells, as measured in the co-culture supernatant when compared to GC-Tfh and GC-B cells cultured in the absence of ILC_FR_ (Fig. 4a). The reduction in IgG levels also demonstrates ILC_FR_ are not a B cell population. Further analysis of co-culture supernatants revealed a significant decrease in secretion of IL-21 by GC-Tfh cells in the presence of ILC_FR_ (*p* < 0.05), but not Tregs (Fig. 4b). We also observed a significant decrease (*p* < 0.01) in sCD40L in the supernatant, when ILC_FR_, but not Tregs, were added to the co-culture of GC-Tfh and GC-B cells (Fig. 4c), demonstrating that this ILC_FR_ subset is able to attenuate GC-Tfh/GC-B cell activation. This decrease in sCD40L could be due to decreased production or increased binding to CD40 on ILC_FR_ to remove it from the microenvironment. Further studies to assess this mechanism should be undertaken. As ILC_FR_ can be found in the follicular areas of the germinal centers, a highly regulated cellular area, indicating they may have an important role to play within the germinal center itself. Collectively, these data strongly demonstrate that ILC_FR_ contribute to disrupting GC-Tfh and GC-B cells interactions, events that occur mainly in the light zone of the germinal center where we have determined follicular ILC_FR_ are present (Fig. 3d–e). Additionally, ILC_FR_ show specific suppressive activity as there were no significant changes in other chemokines/cytokines including BLC (CXCL13), IL-6, IL-8, IL-17, IL-22, MCP-1, IFN- γ, IP-10, MIP-1$\alpha$, MIP-1β, or GM-CSF in the co-culture supernatants after five days with GC-Tfh and GC-B cells with or without Tregs or ILC_FR_ (Supplementary Fig. 6a-k, respectively). Furthermore, there were no significant changes in co-stimulatory surface markers expression on either GC-Tfh (PD-1, ICOS, CD40L) (Supplementary Fig. 7a, 7c, 7e, respectively) or GC-B cells (PD-1, ICOSL, CD40) (Supplementary Fig. 7b, 7d, 7f, respectively) after the five days in co-culture. Of note, there was very little IgG production detected in wells supplemented with 30,000 GC-Tfh or 30,000 GC-B alone in the presence of SEB (Supplementary Fig. 6o). This is due to the necessity of the GC-Tfh/GC-B cell interaction for B-cell-derived IgG production. Of note, it has been previously established that ILC3s participate in enhancing T-cell-mediated immune responses^41,42^. In an attempt to evaluate their biological effect on GC-Tfh-GC-B cell interactions, we co-cultured in vitro ILC3s with GC-Tfh and GC-B cells. Interestingly, ILC3s showed the opposite effect of ILC_FR_ when co-cultured with the GC-Tfh and GC-B cells, leading to significant increases in IgG production (*p* < 0.05) from GC-B cells (Fig. 4d). Given that ILC3s are located outside of the germinal center and express low Bcl6 levels (Fig. 2c), these observations suggest ILC3 may exert an effect on the GC-Tfh-GC-B cell interaction facilitating higher B-cell-derived IgG production. Thus, this newly described ILC_FR_ population is functionally distinct from ILC3s, demonstrating strong suppressive activity on antibody production during the adaptive immune response. These results indicate that ILC_FR_ disrupt the interactions between GC-Tfh and GC-B cells, resulting in the production of less sCD40L and IL-21 by GC-Tfh cells and decreased IgG production by GC-B cells.

### ILC_FR_ produce TGF-β disrupting GC-Tfh/GC-B interactions

**Fig. 5:**
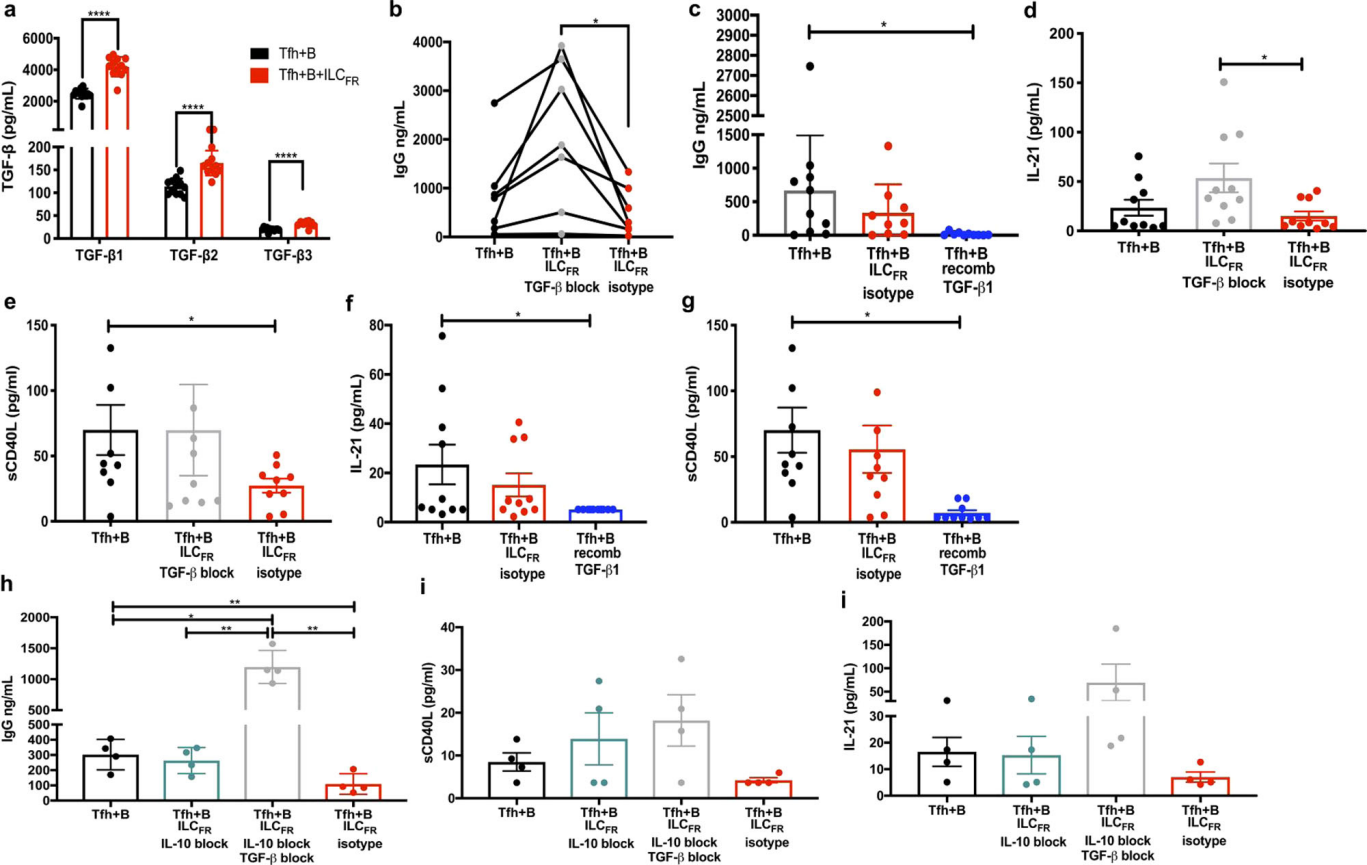
ILC_FR_-derived TGF-β attenuates the interaction of GC-Tfh and GC-B cells and reverses with blockade. **a** Production of TGF-β in the supernatants of 5-day co-culture: 30,000 GC-Tfh with 30,000 autologous GC-B cells (black) plus or minus addition of 1000 ILC_FR_ (red) plus SEB measured by Luminex (*n* = 12 biologically independent tonsils per group, 3 independent experiments) (Paired, two-tailed parametric *t*-test; ****p < 0.0001; df = 11, β1: 95% CI: (1352 to 2048), *t* = 10.76, β2: 95% CI: (32.68 to 69.68), *t* = 6.09, β3: 95% CI: (9.947 to 15.43), *t* = 10.18; Mean ± SD). **b** IgG production in the supernatants of 5-day co-culture with or without blocking of TGF-β with 1ug/mL TGF-β neutralizing antibody (gray) or 1ug/mL mouse IgG isotype control (red) measured by ELISA (*n* = 7 biologically independent tonsils per group, 3 independent experiments) (Ordinary one-way ANOVA with multiple comparisons; F = 8.380; *p = 0.0420; Mean ± SD). **c** IgG production in the supernatants of 5-day co-culture plus or minus 3000 pg/mL of recombinant human TGF-β1 (blue) or 1ug/mL mouse IgG isotype control (red) measured by ELISA (*n* = 10 tonsils per group, 4 independent experiments) (Ordinary one-way ANOVA with multiple comparisons; F = 3.658; *p = 0.0309; Mean ± SD). **d** Production of IL-21 and **e** sCD40L in supernatants of 5-day co-culture with or without ILC_FR_ plus or minus TGF-β neutralizing antibody (gray) or isotype control (red) analyzed by 28 plex Luminex (*n* = 10 biologically independent tonsils per group, 4 independent experiments) (Ordinary one-way ANOVA with multiple comparisons; IL-21: F = 3 = 4.131; *p = 0.0287; sCD40L: F = 3.446; *p = 0.0415; Mean ± SD). (Paired, two-tailed parametric *t*-test; df = 9, sCD40L: *p = 0.0373, 95% CI: (−27.98 to −1.067), *t* = 2.44; IL-21: Tfh+B vs block: **p = 0.01, 95% CI: (9.346 to 51.09), *t* = 3.28, block vs isotype: *p* = .005, 95% CI: (−62.17 to −14.86), *t* = 3.68; Mean ± SD). **f** Production of IL-21 and **g** sCD40L in supernatants of 5-day co-culture with or without ILC_FR_ plus or minus recombinant TGF-β1 (blue) or isotype control (red) analyzed by 28 plex Luminex. (*n* = 10 tonsils per group, 4 independent experiments) (Ordinary one-way ANOVA with multiple comparisons; IL-21: F = 3.654; *p = 0.0394; sCD40L: F = 5.206; *p = 0.0127; Mean ± SD). **h** IgG production in the supernatants of 5-day co-culture plus or minus 1000 ILC_FR_ with or without IL-10 blocking antibody (teal), or both IL-10 and TGF-β blocking antibodies (gray), or isotype control (red) B measured by ELISA. (Ordinary one-way ANOVA with multiple comparisons; F = 43.22; Tfh+B vs. IL-10 and TGF-β block: *p = 0.0325; Tfh+B vs. isotype: **p = 0.0197; IL-10 and TGF-β block vs. isotype: *p* = 0.0114; IL-10 block vs. IL-10 and TGF-β block: *p* = 0.0175; Mean ± SD). **i** Production of sCD40L and **i** IL-21 in the supernatants of 5-day co-culture plus or minus 1000 ILC_FR_ with or without IL-10 blocking antibody (teal), or both IL-10 and TGF-β blocking antibodies (gray), or isotype control (red) measured by ELISA. (*n* = 4 biologically independent tonsils per group, 2 independent experiments).

To further elucidate the mechanism behind the suppressive effects of ILC_FR_ observed in our in vitro co-cultures, we examined whether regulatory components were involved in the disruption of interactions between GC-Tfh and GC-B cells. Based on previous findings demonstrating that intestinal ILC2_10_ exerted their regulatory effects via IL-10 production^3,4^, we performed ELISA and Luminex assays to assess IL-10 production. Interestingly by ELISA, IL-10 was not increased when GC-Tfh and GC-B cells were co-cultured with ILC_FR_ (Supplementary Fig. 8a), also confirmed by Luminex (Supplementary Fig. 8b) as previously reported^3,4^. This led us to examine other potentially suppressive cytokines such as TGF-β in these co-culture supernatants. Strikingly, when compared to the positive control cultures of GC-Tfh and GC-B cells alone, levels of all three subtypes of TGF-β were significantly increased in the presence of ILC_FR_. Indeed, there were significant increases in TGF-β1, 2, and 3 compared to co-cultures without ILC_FR_ (*p* < 0.0001) (Fig. 5a) and cultures with Tregs (*p* < 0.0001) (Supplementary Fig. 6l). Furthermore, stimulation of ILC_FR_ with 1 ug/ml of SEB for 24 h also confirmed that ILC_FR_ are able to produce TGF-β upon activation (Supplementary Fig. 6m, 6n). ILC_FR_ appear to upregulate the expression of the TGF-β1 pathway specifically upon activation as they express low levels of TGF-β1 RNA transcript in their baseline inactivated state (Fig. 2h). We then determined whether the presence of TGF-β was indispensable for suppression of the GC-Tfh cell helper program by assessing the effect of TGF-β neutralization with antibody 1D11. 1D11 is a humanized form of monoclonal antibody that neutralizes the three major active TGF-β isoforms (TGF-β1, -2, and -3), and does not bind other ligands in the TGF-β superfamily, such as multifunctional growth factors activin or bone morphogenetic proteins (BMPs)^43^. When blocking antibody was added daily to co-cultures, we observed significant restoration of GC-Tfh-cell-dependent B cell IgG production (average of 1500 ng/mL increase in IgG and *p*-value < 0.05) in the presence of ILC_FR_. These levels were above those seen in wells with GC-Tfh and GC-B cells only due to complete loss of this suppressive cytokine, and this contrasted with lack of effect upon addition of the isotype control to co-cultures of GC-Tfh and GC-B cells with ILC_FR_ (Fig. 5b). As there were increased levels of TGF-β in the GC-Tfh-GC-B cell control wells, further mechanistic pathway studies blocking other signaling proteins (SMADs) within the TGFβ pathway are warranted. We also determined which subtype of TGF-β was most important for ILC_FR_-mediated suppression of the germinal center reaction, and whether the suppressive cytokine alone was able to disrupt the GC-Tfh-GC-B cell interaction. Supplementing the GC-Tfh-GC-B co-culture with 3000 pg/ml (amount determined in co-cultures after five days with GC-Tfh, GC-B, and ILC_FR_ in the presence of SEB) of exogenous rhTGF-β1, caused a significant reduction in IgG levels (*p* < 05; 23.5-fold decrease), suggesting modulation of GC-Tfh cell function (Fig. 5c), even more significantly than cultures of GC-Tfh and GC-B cells with ILC_FR_. These data demonstrate that TGFβ-1 alone is sufficient to recapitulate the suppression observed when ILC_FR_ are present. This effect was not due to compound-related toxicity, as shown by viability measurement after co-culture assays using flow cytometry (Supplementary Fig. 7g, 7h). Cytokine analysis of co-culture supernatants at day 5 demonstrated the effect of TGF-β on IL-21 production by GC-Tfh cells. Blocking TGF-β caused levels of IL-21 to increase to levels higher than those observed in cultures with GC-Tfh and GC-B cells with ILC_FR_ plus isotype controls (*p* < 0.01; Fig. 5d). However, comparing ILC_FR_ plus isotype wells to the GC-Tfh and GC-B cell positive control showed no significant difference in IL-21 or IgG levels (Fig. 5b, d). This highlights the importance of the TGF-β pathway in modulating the interactions of ILC_FR_ with GC-Tfh and GC-B cells. TGF-β blockade, however, had no effect on sCD40L secretion (Fig. 5e). Additionally, supplementation of rhTGF-β1 to cultures of GC-Tfh and GC- B cells showed the most significant inhibition of IL-21 (*p* < 0.05; Fig. 5f) and sCD40L levels in the culture supernatants (*p* < .01; Fig. 5g), which indicates TGF-β1 is able to most effectively suppress GC-Tfh cell help and subsequent GC-B cell function. Interestingly, RNAseq data showed that ex vivo sorted GC-Tfh cells expressed higher level of *TGFR1* and *TGFR2* compared to GC-B cells (Table 1), suggesting that GC-Tfh cells may be preferentially impacted by TGF-β secretion from ILC_FR_ in the co-culture. To determine whether IL-10 produced by ILC_FR_ could also interfere with GC-Tfh-GC-B cell interaction, we performed IL-10 neutralization experiments in the co-culture assays. Addition of an IL-10 neutralizing antibody in co-cultures containing ILC_FR_ failed to recover IgG production (Fig. 5h). Furthermore, there was no recovery of sCD40L (Fig. 5i) or IL-21 levels (Fig. 5i) with IL-10 neutralization. Statistically significant increases in IgG levels were only observed when TGF-β neutralization was present (Fig. 5h). Based on these findings, our data indicates that TGF-β production from these newly described ILC_FR_ are responsible for the majority of the suppression observed of the GC-Tfh-GC-B cell interaction.

**Table 1:** Cytokine Receptor expression on sorted human tonsillar cell subsets.

	ILC1	ILC2	ILC3	ILC_FR_	GC-Tfh	GC-B	Treg
IL-7R	403.6734	237.6888	398.1677	70.21166	31.58694	0.0718	79.08643
IL15RA	4.173651	5.759125	4.736666	4.838582	0.902477	3.679552	12.40327
IL-15	1.220763	1.341808	0.879143	1.736831	0	0	0.296501
TGFBR1	9.13155	5.65353	8.35876	3.86731	15.65845	7.51772	8.86576
TGFBR2	64.4227	45.61228	68.33044	36.08439	45.3508	22.28398	48.92418

RPKM values were used to show IL-15 expression, TGF-βR, IL-7R, and IL-15R receptor expression on tonsillar sorted subtypes with this table.

### ILC_FR_ are expanded during chronic HIV infection

**Fig. 6:**
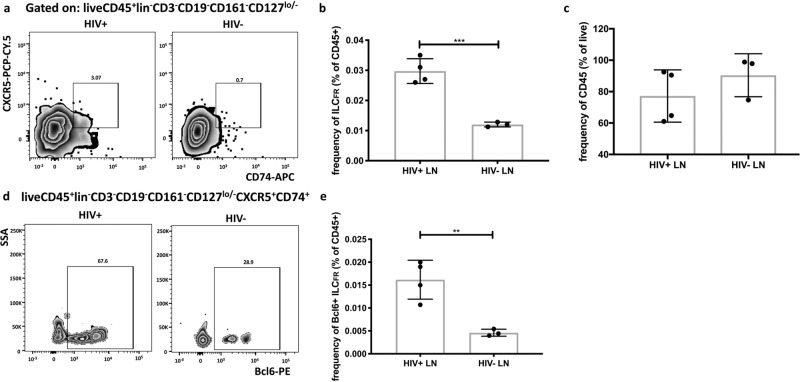
ILC_FR_ are expanded in chronic HIV viral infection in human lymph nodes. In **a** plots shows frequency of ILC_FR_ in human HIV^−^ or chronic infection HIV^+^ lymph nodes. Gated on live cells, CD45^+^lin^−^CD3^−^CD19^−^CD161^−^CD127^lo/−^. In **b** the graph shows frequency of ILC_FR_ among CD45^+^ lymph node cells in both HIV^−^ and HIV^+^ individuals (unpaired, two-tailed nonparametric *t*-test; ***p = 0.0008; df = 5, 95% CI: (−0.02406 to −0.01140), *t* = 7.198; Mean ± SD). **c** shows frequency of total live CD45^+^ cells in both groups from **b**. In **d** the plots show Bcl6 expression in ILC_FR_ in human HIV^−^ or HIV^+^ lymph nodes, gated on CD45^+^lin^−^CD3^−^CD19^−^CD161^−^CD127^lo/−^CXCR5^+^CD74^+^ from the gates in **a**, and **e** shows the graph of frequency of Bcl6^+^ among CD45^+^ lymph node cells in HIV^−^ and HIV^+^ individuals (unpaired, two-tailed nonparametric *t*-test; **p = 0.0061; df = 5, 95% CI: (−0.01809 to −0.00504), *t* = 4.559; Mean ± SD) (2 independent experiments) (*n* = 3 or 4 biologically independent tonsils per group).

We examined the dynamics of ILC_FR_ in the context of HIV infection. The hallmarks of HIV infection include a persistent systemic inflammatory state and immune dysfunction^44,45^ as well as overwhelming TGF-β-induced fibrosis of lymph nodes that is only partially restored by antiretroviral therapy^46^. Thus, we hypothesized that ILC_FR_ may be altered in this disease state. In fact, we and others have previously shown that GC-Tfh cells from HIV^+^ lymph nodes are functionally impaired in their interactions with GC-B cells despite their increase in cell numbers^37,47^. This defect in GC-Tfh cells could contribute to the altered quality of anti-HIV antibody responses during infection. However, the underlying mechanisms of this cellular dysfunction remain poorly understood. We found a significant increase in frequencies of ILC_FR_ among lymph nodes cells of HIV^+^ compared to HIV^−^ individuals (*p* < 0.05; Fig. 6a, b), whereas there was no change in overall numbers of CD45^+^ lymph node cells (Fig. 6c). Additionally, expression of Bcl6, a transcription factor required for germinal center entry^13^, was significantly higher (*p* < 0.01) among the expanded ILC_FR_ population in HIV^+^ compared to HIV^−^ individuals (Fig. 6d, e), suggesting that ILC_FR_ are not only expanded in HIV infection, but are also more likely to be located within the germinal center follicle compared to in the absence of infection. Taken together, these data indicate that this ILC_FR_ cell population may be contribute to the immune dysfunction associated with chronic HIV infection.

## Discussion

Here we describe an ILC population that we termed follicular regulatory innate lymphoid cells (ILC_FR_), which excitingly appear to play an important role in disrupting the interactions between GC-Tfh and GC-B cells within germinal centers. We demonstrate that ILC_FR_ are present and functional in human tonsils and lymph nodes where we show that they were localized within the germinal center follicle and are uniquely identified by CD74, and ID3 expression. While these cells express some B cell RNA transcripts such as CD19 and EBF1, they are not B cells, T cells or ILC1s, ILC2s, or ILC3s based on RNA seq and MDS plot analysis. ILC_FR_ are negative for CD19 protein expression via flow cytometric experiments and were gated stringently as CD19 negative in addition to other cell lineage markers. Furthermore, we see decreased IgG in T cell–B cell co-cultures in the presence of ILC_FR_. ILC_FR_ can be observed between B cells and ILCs on the MDS plot, closer to ILC2s and ILC3s, and they demonstrate more expression of common markers and transcription factors with ILCs (ID2, CD7, CCR6, IL2RG, IL12RB1) than they do B cells or T cells. Additionally, while ILC_FR_ share some low levels of common ILC1 transcripts (Tbet), or dendritic cells, combining extensive gene transcript analysis for all markers for each lineage and functional assays of cytokine and activation shows that ILC_FR_ are in fact unique from these cell populations. Furthermore, these cells are not ILC2s, as they lack GATA3, KLRG1, and other ILC2 necessary markers. Furthermore, in contrast to the newly described ILC2_10_ cells, which employ the IL-10 program for immunosuppression, ILC_FR_ produce robust amounts of TGF-β upon activation, and can suppress GC-Tfh cell-dependent IgG production from GC-B cells, thus disrupting their interaction. Co-culture of GC-Tfh and GC-B cells with ILC_FR_ also leads to decreased IL-21 and sCD40L production in vitro, both known to be necessary for germinal center formation and antibody secretion. Further studies into whether ILC_FR_ directly contact the GC-Tfh or GC-B cells to exert their immunosuppressive effects are warranted. RNA-sequencing data and functional analysis suggests that ILC_FR_ likely sense inflammatory microenvironments through inflammatory cytokine receptors (CD74, IFNGR1 and 2, IFNAR1 and 2, IL17RA), are activated, and begin to secrete TGF-β to exert their immunosuppressive effects. Hence, in chronic states where there are high levels of inflammatory cytokines (such as chronic HIV), ILC_FR_ may be expanded in attempts to produce more TGF-β. Overall, this data supports the hypothesis that these novel cells are unique from other defined T, B and ILC subsets, and are involved in disrupting T cell–B cell interactions in the germinal center, which are critical for an efficient humoral response in our hands. It is possible that ILC_FR_ serve to counterbalance the T cell–B cell interaction in the germinal center by limiting the IL-21 and sCD40L pathways, crucial for germinal center formation and maintenance. Overall, our findings identify a unique innate lymphoid subset with distinct phenotype, function and location. Our data further demonstrate that these cells can regulate initiation and development of immune responses in health and disease.

It has also been established that germinal center reactions are impaired in chronic viral infections and autoimmune diseases^23,48^. The hallmarks of HIV-1 pathology include immunodeficiency, lymphoid tissue destruction, gut barrier breakdown, and systemic immune activation^44^. These features are only slightly reversed by viral suppressive long-term antiretroviral therapy^49^. The cause of the progressive depletion of CD4^+^ T cells in HIV-infected individuals is one of the most fundamental and controversial issues in HIV/acquired immunodeficiency syndrome (AIDS)^50^ research. Additionally, previous studies have suggested that lymph node fibrosis following HIV infection is an important mechanism underlying the pathogenesis of HIV/AIDS due to the necessity of healthy lymph node tissue for maintenance of immune homeostasis as well as the survival, proliferation and differentiation of lymphocytes^51,52^. Furthermore, TGF-β1 is considered to serve a central role in inducing fibroblasts to synthesize collagen and thereby induce fibrosis^53^. As our data showed a 3-fold increase in the frequency of ILC_FR_ in chronic HIV-1 infection, we hypothesize that this increase in ILC_FR_ may lead to an increase in the amount of TGF-β in the germinal center and elicit fibrotic reactions. Therefore, ILC_FR_ may be an important source of the TGF-β found in secondary lymphoid organs in HIV infection.

Finally, the tumor microenvironment, as with the immunological milieu of chronic infection, contains a multitude of suppressive mechanisms that allow tumors to escape immune surveillance similar to virally infected cells in chronic viral infections. Both of these chronic inflammatory states lead to dysfunctional adaptive T cell responses^54^. Therefore, expanding our ILC_FR_ characterization into cancer models will be an interesting natural next step based on the compelling chronic HIV infection data. Many cancers have inactivating mutations in their TGF-β receptor^55,56^ or SMAD complex pathways^57^, discontinuing the tumor suppressive effects of TGF-β signaling especially in cells already harboring oncogenic mutations (premalignant). Furthermore, the presence of tumor-infiltrating lymphocytes coincides with the highest levels of TGF-β secretion and is thus a suspected source of the accumulation of TGF-β1 in the tumor microenvironment^58^. While the specific cells producing TGF-β remain elusive, we propose that with the understanding gained about ILC_FR_, these cells may be an as yet unidentified source of this suppressive cytokine that can lead to cancer progression and immune dysregulation. ILC_FR_ are trafficked to sites of inflammation such as those seen in chronic HIV-infected lymph nodes, are activated by inflammatory microenvironments, produce large amounts of TGF-β, expand in frequency, and nonspecifically suppress the chronic inflammatory state seen in viral infections and cancer microenvironments. We believe these newly identified ILC_FR_ could play a key role in the dysfunction seen in both viral and tumor microenvironments.

In conclusion, ILC_FR_ are activated by inflammatory environments and exert their suppressive function on GC-Tfh and GC-B cell interactions. They disrupt interaction of GC-Tfh and GC-B cells in the germinal center by secreting large amounts of TGF-β which leads to decreased sCD40L, IL-21, and IgG production. This loss of IgG production illustrates the inability of GC-Tfh cells to appropriately help the GC-B cells respond to antigen. This could lead to diminished vaccine responses and reactions to infection. The expansion of this suppressive cell population observed in chronic HIV infection could also help explain the immune dysfunction and collagen deposition (fibrosis) that are hallmarks of this disease. Given that tumor microenvironments and autoimmune diseases are also characterized by a defective adaptive immune response, there is perhaps a role for ILC_FR_ in these disease contexts. Further studies are needed to investigate the role that ILC_FR_ may play in many diseases of immunologic dysfunction and how targeting them could serve as a novel therapeutic intervention.

## Methods

### Lead contact

Further information and requests for resources and reagents should be directed to and will be fulfilled by the Lead Contact, Elias Haddad (ee336@drexel.edu).

### Human samples

Blood samples and tonsils obtained from donors with repeated chronic tonsillar infections but were uninfected at time of tonsil removal at both Martin Memorial Health Systems (Florida) and St. Christopher’s Hospital for Children (Pennsylvania). Tonsils used were from patients aged 2–45, and were digested, made into single cell TMNC suspensions and frozen in bovine serum albumin (Sigma) plus 10% DMSO (VWR) for cryopreservation in liquid nitrogen. The Institutional Review Boards at the relevant institutions approved all procedures, and all participants provided signed informed consent. The Drexel University College of Medicine’s Institutional Review Board approved the pediatric tonsil collection with a waiver of consent process (IRB#1808006563) and collection was at St. Christopher’s Hospital for Children (Pennsylvania).

Biopsies of palpable inguinal, cervical or axillary lymph nodes were performed at the National Institutes of Health Clinical Research Center in Bethesda, Maryland under protocols approved by the NIAID Institutional Review Board (ClinicalTrials.gov Identifier: NCT00001316). All participants provided written informed consent. The lymph node samples were obtained from individuals with varying levels of plasma HIV viremia, ranging from 700 to 300,000 viral copies/mL, and with CD4 counts ranging from 220 to 470 cells/mm^3^. See Table 2 detailing the HIV+ and HIV− patients’ immune status.

**Table 2:** HIV+ and HIV− patients’ immune status.

HIV-NEG	Date	LNMC count	VL	CD4	%CD4	CD8	Sex	Age	Excision LN		
HD KerBen	12/31/2018	50 M	NA	919	55	284	Female	30	Left inguinal		
HD BM	6/8/2016	5.5 M	NA	1140	50	593	Male	45	Left inguinal		
HD E5	7/6/2016	23 M	NA	856	47	346	Female	26	Right inguinal		

HIV-POS	Date	LNMC count	VL	CD4	%CD4	CD8	Sex	Age	Excision LN	ARV status	HIV status
MC	4/18/2005	120 M	742	471	8	4765	Male	39	Right inguinal	ART for 1 M	Early/chronic: >4 months
ED	1/19/2006	10 M	12,743	422	23	991	Male	20	Right axillary	No ART	Chronic: 1–2 years
JB	3/9/2006	20 M	221,158	369	18	819	Male	44	Right inguinal	No ART	Chronic: ~6 months
EK	7/29/2009	50 M	293,361	220	10	1628	Male	29	Left axillary	No ART	Chronic: unknown, latent TB

HIV positive and negative human lymph node details. These include viral load, lymph node mononuclear cell counts, CD4/CD8 T cell counts, patient age, gender, and status of retroviral therapy and HIV disease.

### Phenotypic analysis of germinal center

Tonsil cells from adult donors or HIV^+^ lymph nodes were incubated with fluorochrome-conjugated antibodies. The following fluorochrome-conjugated antihuman antibodies were used: CD3 (Clone: HIT3α, Cat: 300324), CD4 (Clone: RPA-T4, Cat: 300520), CD25 (Clone: BC96, Cat: 302608), CD38 (Clone: HIT2, Cat: 303524), PD-1 (Clone: EH12.2H7, Cat: 329924), PD-L1 (Clone: 29E.2A3, Cat: 329708), CD319 (Clone: 162.1, Cat: 331806), CD26 (Clone: BA5b, Cat: 302706), ICOS (Clone: C398.4A, Cat: 313506), CD11c (Clone: BU15, Cat: 337110) CD11b (Clone: LM2, Cat: 393110), CD16 (Clone: 3G8, Cat: 302026), CD14 (Clone: M5E2, Cat: 301822), CD161 (Clone: HP-3G10, Cat: 339916), NKp44 (P44-8, Cat: 325110), CCR6 (Clone: G034E3, Cat: 353404), IL-10 (Clone: JES3-9D7, Cat: 601420), TGF-β1 (Clone: TW4-2F8, Cat: 349610), CD40 (Clone: 5C3, Cat: 334308), CD40L (Clone: 24-31, Cat: 310810), IL2RG (Clone: TUGm2, Cat: 132305), CD7 (Clone: CD7-6B7, Cat: 343118), CD74 (Clone: LN2, Cat: 326812), CD56 (Clone: 5.1H11, Cat: 362504), KLRG1 (Clone: 14C2A07, Cat: 368614) were all from BioLegend. IL12RB1/CD212 (Clone: 2-4E6, Cat: 556065), CD294/CRTH2 (Clone: BM16, Cat: 563501), Tbet (Clone: 04-46, Cat: 561268), GATA3 (Clone: L50-823, Cat: 560068), CD19 (Clone: HIB19, Cat: 557921), CXCR5 (Clone: RF8 B2, Cat: 356910), IgD (Clone: IA6-2, Cat: 348226) and Bcl6 (Clone: K112-91, Cat: 561522) ICOSL (Clone: 2D3/B7-H2, Cat: 309403), CD39 (Clone: TU66, Cat: 560239), CD45 (Clone: 2D1, Cat: 560178), CD117 (Clone: YB5.B8, Cat: 559879), CD127 (Clone: A019D5, Cat: 351310), ID3 (Clone: S30-778, Cat: 564564), RORγt (Clone: Q21-559, Cat: 563081) were from BD Biosciences and CD45RA (Clone: 2H4LDH11LDB9, Cat: IM2711U) from Beckman Coulter, FoxP3 (Clone: PCH101, Cat: 53-4776-42) from eBioscience. LIVE/DEAD Fixable Dead Cell Stain (Life Technologies, Cat: L34957) was used to gate on live cells. Cells were phenotyped as follows: ILC1s were Lineage^−^(CD19^−^CD16^−^CD14^−^CD4^−^CD11c^−^CD11b^−^)CD3^−^CD45^+^CRTH2^−^CD127^+^CD161^+^CD117^−^, ILC2s as Lineage^−^CD3^−^CD45^+^CRTH2^+^CD127^+^CD161^+^, ILC3s as Lineage^−^CD3^−^CD45^+^CRTH2^−^CD127^+^CD161^+^CD117^+^, ILC_FR_ as Lineage^−^CD45^+^CD127^lo^CD161^−^CD74^+^ID3^+^CXCR5^+^, Treg as CD3^+^CD4^+^CD45RA^−^CXCR5^−^PD1^−^CD25^+^CD127^lo^ GC-Tfh as CD4^+^CD3^+^CD45RA^−^CXCR5^hi^PD1^hi^, GC-B as CD19^+^CD38^int^IgD^−^CD319^lo^. Samples were acquired on a BD^TM^ FACS Aria.

### Functional analysis of germinal center

Tonsil cells from healthy donors were stimulated with 1 ug/mL of staphylococcal enterotoxin B (SEB) (Toxin Technology) with Golgi stop (Invitrogen) in RPMI 1640 (Corning) with 10% fetal bovine serum (Access Biologicals) and 1% penicillin/streptomycin (Gibco) for 6 h before the staining protocol outlined above was employed or for 24 h before TGF-β1 production was assessed.

### Co-culture assay

Tonsils from donors were thawed and then incubated with fluorochrome-conjugated antibodies for 15 min at 4 °C in the dark. Samples were sorted on a BD^TM^ FACSFusion. Germinal center Tfh cells were CD3^+^CD4^+^CD45RA^−^CD25^−^CXCR5^hi^PD-1^hi^; germinal center B cells were defined as CD19^+^IgD^−^CD38^+^CD319^lo^; tonsil CD4 Tregs defined as CD3^+^CD4^+^CD45RA^−^CD127^lo^CD25^+^CXCR5^lo^PD-1^lo^; ILC3 defined as Lineage^−^(CD19^−^CD16^−^CD14^−^CD4^−^CD11c^−^CD11b^−^) CD3^−^CD45^+^CRTH2^−^CD127^+^CD161^+^CD117^+^; ILC_FR_ defined as Lineage^−^CD3^−^CD45^+^CD127^+^CD161^−^CD74^+^. Sorted CD4 Treg, ILC_FR_, and ILC3 were plated for co-culture with autologous GC-B cells and GC-Tfh at either equal ratio or with cell counts specified with 100 ng/mL of staphylococcal enterotoxin B (SEB) (Toxin Technology) in RPMI 1640 (Corning) with 10% fetal bovine serum (Access Biologicals) and 1% penicillin/streptomycin (Gibco). Alternatively, some co-culture assays were supplemented daily for 5 days with either 1ug/mL of TGFβ-1,2,3 monoclonal blocking antibody (1D11) (ThermoFisher, Cat: MA5-23795), plus or minus 1 pg/mL of Rabbit Anti-Human IL-10 blocking antibody (Peprotech, Cat: 500-P20), with 1 pg/mL of rabbit control immunoglobulin isotype control for IL-10 cultures (Peptrotech, Cat: 500-P00) or 1 ug/mL mouse IgG1 isotype control (ThermoFisher, Cat: 02-6100) for TGFβ-1,2,3 supplemented culture control. For rhTGF-β1 (R&D Systems; Cat: 240-B-002) supplemented wells, 3000 pg/mL of rhTGF-β1 was once added at day 0 to the experimental wells or 1ug/mL mouse IgG isotype control. Cells were kept for 5 days (tonsil) in co-culture followed by flow cytometry analysis. Supernatant and cells were collected at day 5 for subsequent analysis.

### Total IgG and IL-10 ELISA

For human samples, total IgG was measured by ELISA on culture supernatant as previously described^40^. Total IgG was detected by coating 96-well Immulon 2HB plates (Thermo Fisher Scientific) with antihuman monoclonal IgG (Mabtech, clone MT91/145) at a concentration of 1 μg/mL in phosphate buffered saline (PBS) overnight at 4 °C or antihuman IL-10 (ThermoFisher Invitrogen 4311238). The next day plates were washed three times with wash buffer (PBS + 0.05% Tween 20), and subsequently left to block with wash buffer for 1 h at room temperature. Plates were then washed before the addition of sample and IgG standards at different dilutions, for 2 h at room temperature. For IL-10 ELISA, the IL-10 standards were used. Following sample incubation and washing, the plates were left to incubate with 1 μg/mL of antihuman IgG-biotin (Mabtech, clone MT78/145) for 1 h at room temperature or antihuman IL-10 biotin (ThermoFisher Invitrogen Cat: 4309921). The wash step was repeated, and the plates incubated with streptavidin-HRP (Mabtech for IgG or ThermoFisher 4337572 for IL-10) for 1 h at room temperature. An extra wash was added to the last wash step before adding 100 μL of TMB substrate (Sigma–Aldrich) to each well until a color change was observed. The reaction was stopped by the addition of 50 μL of 1 M H_3_PO_4_. The OD values were read at 450 nm using a spectrophotometer (SpectraMax Plus, Molecular Devices).

### Cytokine and chemokine analysis

Supernatants collected from tonsils in co-culture were analyzed for chemokine/cytokine levels using Bio-Plex Pro magnetic bead assays (Bio-Rad, Hercules, CA USA). The following human chemokine premixed panels was used: BLC (CXCL13), sCD40L (sCD154), MCP-1 (CCL2), MIP-1α (CCL3), MIP-1β (CCL4), SDF-1 (CXCL12), MIP-3β (CCL19), MIP-3α (CCL20), GM-CSF, IP-10 (CXCL10), Fractalkine (CX3CL1), IL-1β, IL-2, IL-4, IL-6, IL-8, IL-9, IL-10, IL-12p70, IL-13, IL-15, IL-17A, IL-21, IL-22, IL-23, TNF-α, IFN- α, IFN-γ and TGF-β1, TGF-β2, TGF-β3. The manufacturer’s protocol was followed. Data were acquired on a Bio-Plex 200 System (using bead regions defined in the Bio-Rad protocol) and analyzed with the Bio-Plex Manager 6.1 software from Bio-Rad.

### Gene expression analysis

Healthy human donor tonsil cells (from seven biologically distinct humans) were directly sorted into cold RLT buffer (QIAGEN) supplemented with 1% beta-mercaptoethanol (βM) (Sigma) and quickly stored at −80 °C. The seven cell populations were sorted to 2000–5000 cells each and included GC-Tfh, GC-B, Treg, ILC1, ILC2, ILC3, and ILC_FR_ described phenotypically above. Total RNA was isolated using the RNeasy Micro Kit (Qiagen) following recommended procedures, with on-column DNase treatment. Total RNA was normalized prior to oligo-dT capture and cDNA synthesis with SMART-Seq v4 (Takara). RNA libraries were generated using the Nextera XT DNA Library Prep Kit (Illumina). All sample quality assessment was performed on a 5300 Fragment Analyzer System (Agilent) and quantified using a Qubit 3.0 fluorometer (Life Technologies). Medium depth sequencing (>16 million reads per sample) was performed on a NextSeq 550 System (Illumina) using two High Output flow cells each with a 75 base pair, Paired End run.

Demultiplexed fast-q paired end read adapters of length less than 36 and average phred quality score of less than 30 were trimmed and filtered using the skewer software^59^. Alignment was performed with HISAT2 to the Homo sapiens NCBI reference genome assembly version GRCh38 and sorted with SAMtools^60,61^. The aligned reads were counted and assigned gene meta-information using the featureCounts software^62^, and analysis was conducted using the R programming language and various packages from the Bioconductor suite. Normalization, weighting, and subsequent differential expression was performed using LIMMA^63^ and geneset enrichment analysis was performed using GSVA pathway enrichment sets from MsigDB^64,65^.

### Immunohistocytometry antibodies

Tissues were stained with the following antibodies: *Primary/Conjugated antibodies:* CD74 (Rabbit polyclonal, Abcam, #ab64772), CD8 (Mouse IgG2b, clone: 4B11, Thermo Scientific, #MA1-80231), ID3 (Mouse IgG1, clone: OTI8B3, abcam, ab236505), Ki67 Brilliant Violet 480 (BD Horizon, clone: B56, 566172), CD19 Alexa Fluor 647 (Biolegend, clone: A17136C, 396304), CD4 Alexa Fluor 700 (R&D systems, goat polyclonal, FAB8165N). *Secondary antibodies:* Donkey anti- Rabbit IgG Brilliant Violet 421 (Biolegend, clone: poly4064; 406410), Goat anti-Mouse IgG2b Alexa Fluor 488 (Life Technologies, A21141), Goat anti-Mouse IgG1 Alexa Fluor 594 (Life Technologies, A21125). Mouse IgG1 kappa isotype control clone B11/6, abcam, ab91353.

### Multiplexed confocal imaging

Paraffin tissue blocks were cut into 5-micron-thick sections and subsequently mounted onto slides (Leica-Apex Superior Adhesive Slides). Deparaffinization/rehydration was achieved through sequential Xylene-Ethanol-diH_2_0 solutions. Antigen retrieval via heating/pressurization (110 degrees Celsius/ 5–6 PSI/ 15 min) of the slides was performed with Reveal Decloaker (Biocare Medical) in a decloaking chamber (Biocare Medical). After cooling down the slides, the tissues were blocked/permeabilized with a Phosphate Buffered Solution (PBS)/Bovine Serum Albumin (BSA)/Triton-X solution. Titrated amounts of primary antibodies were then added for overnight staining at 4 °C. Next day, slides were washed in PBS before being incubated with titrated amounts of appropriate secondary antibodies for 2 h, which was followed by conjugated antibodies. A final washing step with PBS was then performed, and then the nuclear stain Jopro-1 was applied (ThermoFisher Scientific). Slide were subsequently mounted with a glass coverslip using Fluoromount G (Southern Biotech).

Confocal images were obtained using a Nikon (A1+) confocal system operated through the NIS-elements AR software. A ×40 (NA 1.3) objective was used to scan the tissues. Multiple ×40 field of views and Z stack slices generated from each tissue were stitched together via the NIS-elements AR software. Pixel density of each field of view was 512 × 512. No frame averaging or summing was used while obtaining the images. Separation of fluorescence emitted from the fluorophores was achieved utilizing the NIS-elements AR software’s “spectral unmixing” function. Briefly, utilizing single fluorophore tissue staining, an emission spectrum database was created, which was subsequently utilized by the program to separate the emission of different fluorophores (simultaneously or sequentially exited) into different channels.

Isotype imaging was on FFPE tonsillar tissue sections that were stained with either a primary isotype control antibody (mouse IgG1 kappa isotype control clone B11/6, abcam, ab91353; or an ID3 specific antibody. Image acquisition was performed on a Leica SP8 confocal microscope with a ×40 objective (NA 1.30) at 1% zoom. Images were visualized using the software Imaris version 9.6.0 (Bitplane). Staining produced a nuclear pattern consistent with transcription factor localization as previously described (https://www.proteinatlas.org/ENSG00000117318-ID3/antibody).

### Quantitative imaging analysis (histocytometry)

Confocal images were analyzed with Imaris software version 9.5.0 (Bitplane). Histocytometry analysis was performed to generate quantitative data from the images. Briefly, 3-dimensional segmented surfaces (based on nuclear signal) of spillover corrected images were generated with Imaris via the Surface Creation module. Average voxel intensities for all channels, as well as volume and sphericity of the 3-dimensional surfaces generated from histocytometry were exported in Microsoft Excel format. After conversion of the files to comma separated value (.CSV) files, the data were imported into FlowJo (version 10) for further analysis/quantification. The results of the histocytometry analysis were expressed as frequencies.

### Statistics and reproducibility

All flow cytometry, co-culture, ELISA and Luminex data were analyzed using GraphPad Prism v7. Paired Student’s *t*-test (Wilcoxon) was used when comparing two groups. The Paired multiple *t*-test and nonparametric one-way ANOVA test was used when comparing more than two groups to each other. (*p < 0.05, **p < 0.01, ***p < 0.001, ****p < 0.0001). N numbers ranged from 4–17 biologically distinct individual patient tonsils per group and included at least two independent experiments per assay.

### Reporting summary

Further information on research design is available in the Nature Research Reporting Summary linked to this article.

## Supplementary information

Supplementary Information

Reporting Summary

## Data Availability

Sequence and gene expression data are available at the Gene Expression Omnibus (accession number GSE168407). All other data are available in the main text or supplementary materials.
