# Supplementary material for: A follicular regulatory Innate Lymphoid Cell population impairs interactions between germinal center Tfh and B cells
Source: Commun Biol. 2021 May 12;4:563. doi: 10.1038/s42003-021-02079-0 (PMC8115650; doi:10.1038/s42003-021-02079-0)
Supplement: Supplementary file 1 — Supplementary Information [file 42003_2021_2079_MOESM1_ESM.pdf]

Supplementary Figures

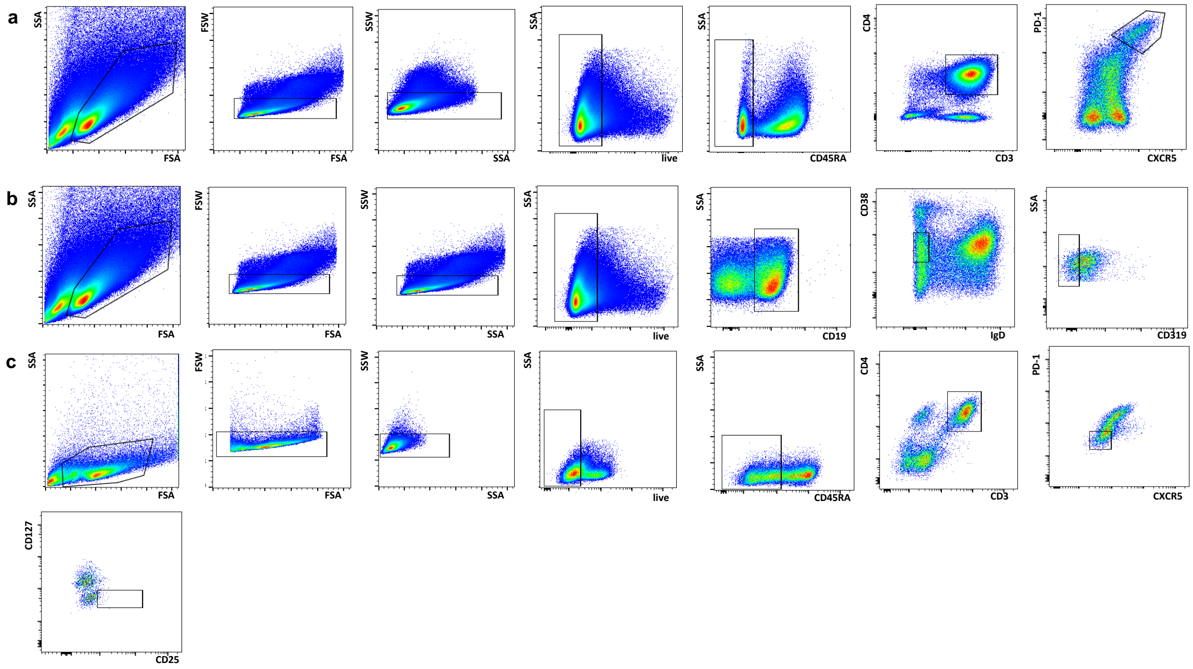

Supplementary Figure 1. Gating Strategy for GC-Tfh, GC-B, Treg

(a) Representative conventional flow cytometry plots from uninfected adult tonsil mononuclear cells showing the hierarchical phenotype staining from singlet lymphocytes to GC-Tfh, (b) GC-B cells, and (c) T regulatory cells (n = 14 biologically independent tonsils per group, 4 independent experiments).

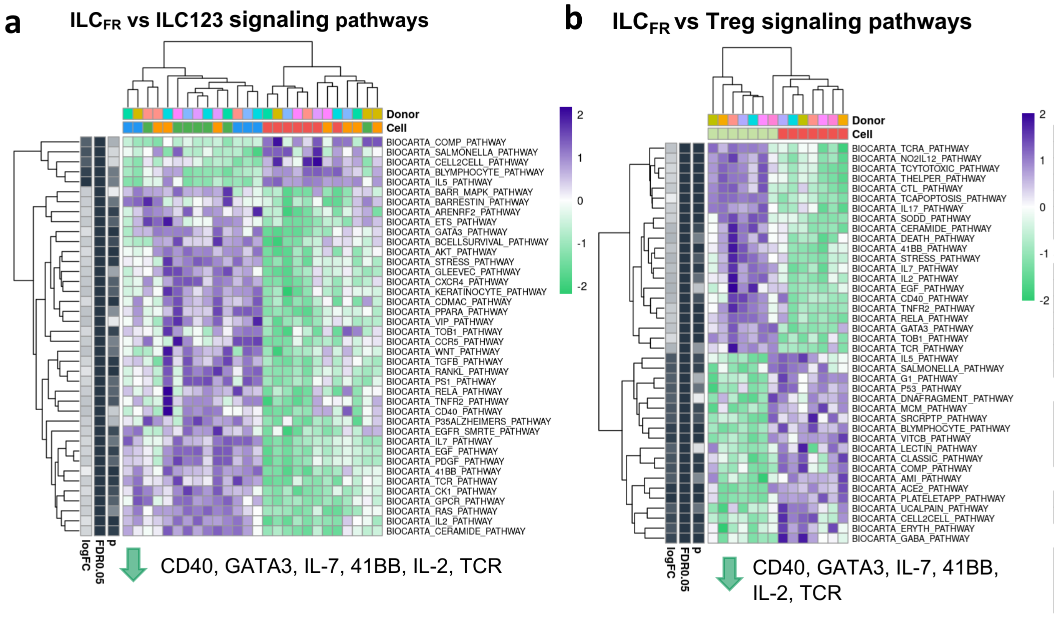

c

	ILC1	ILC2	ILC3	ILC_{FR}	GC-Tfh	GC-B	Tregs
CD19	1.30844486	190.188303	63.6088116	345.215253	3.45156697	520.061186	49.8055351
EBF1	0.06894185	6.14374697	1.67244756	14.7852567	0.466248	27.7587384	1.9611757
CD24	0.03898881	10.2075085	5.57053489	25.5971998	0.33901789	38.7032006	4.28108012
CD40	6.5202147	91.6723741	34.7798375	242.441187	4.91530071	427.402721	26.2830061
CD86	0.08778907	12.1314848	3.14481531	34.9340803	0.48943348	44.4521634	3.28036855

Supplementary Figure 2. ILC_{FR} are unique in signaling and gene expression from ILC123 and Tregs, GC-B, and GC-Tfh

(a) the heatmap shows relative gene expression patterns of canonical signaling pathways comparing ILC_{FR} to ILC1, ILC2, ILC3 and (b) Tregs among $CD45^+$ uninfected human tonsil mononuclear cells (n = 7 biologically independent tonsils per group) (DE paired two tailed T test with Bayes extension for RNA seq). (c) shows a table with RPKM values for antigen presenting cell and B cell markers in all 7 sorted cell populations.

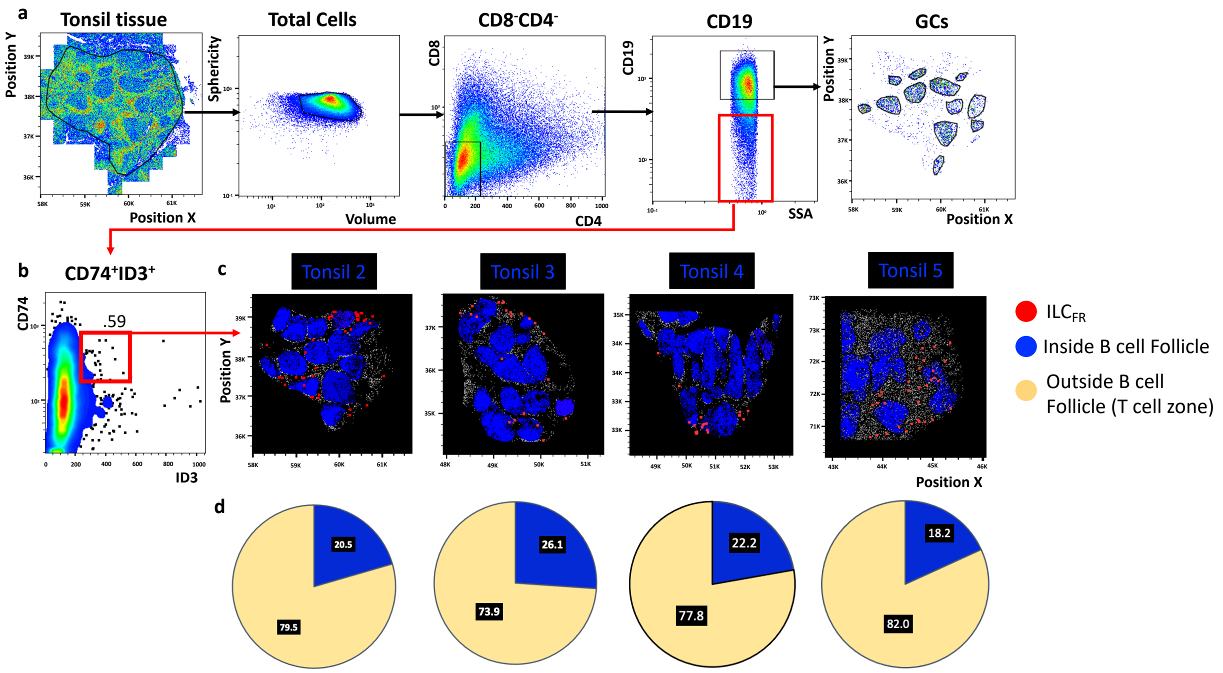

Supplementary Figure 3. Gating strategy used for histocytometry analysis

In (a) is the gating strategy used for the quantitative analysis of confocal images in FlowJo after the application of nuclear marker (JOPRO-1) guided cell segmentation in Imaris. B cell follicles were defined as areas of high CD19 expression ($CD19^{hi}$) whereas ILC_{FR} were defined as $CD8^{-}CD4^{-}CD19^{-}$ cells positive for CD74 and ID3. (b) Representative histocytometry plot showing the frequency of $CD8^{-}CD4^{-}CD19^{-}CD74^{+}ID3^{+}$ (ILC_{FR}) cells in a tonsil tissue section. (c) Overlays projecting 2D distributions of CD74+ID3+ ILC_{FR} (red) in $CD19^{hi/dim}$ areas (B cell follicles; blue) in the 4 other biologically independent human tonsil sections as determined by histocytometry (d) Pie charts showing the percentage of intrafollicular (blue) and extrafollicular ILC_{FR} (beige) as a frequency of total ILC_{FR} for the same 4 tonsils shown in (c).

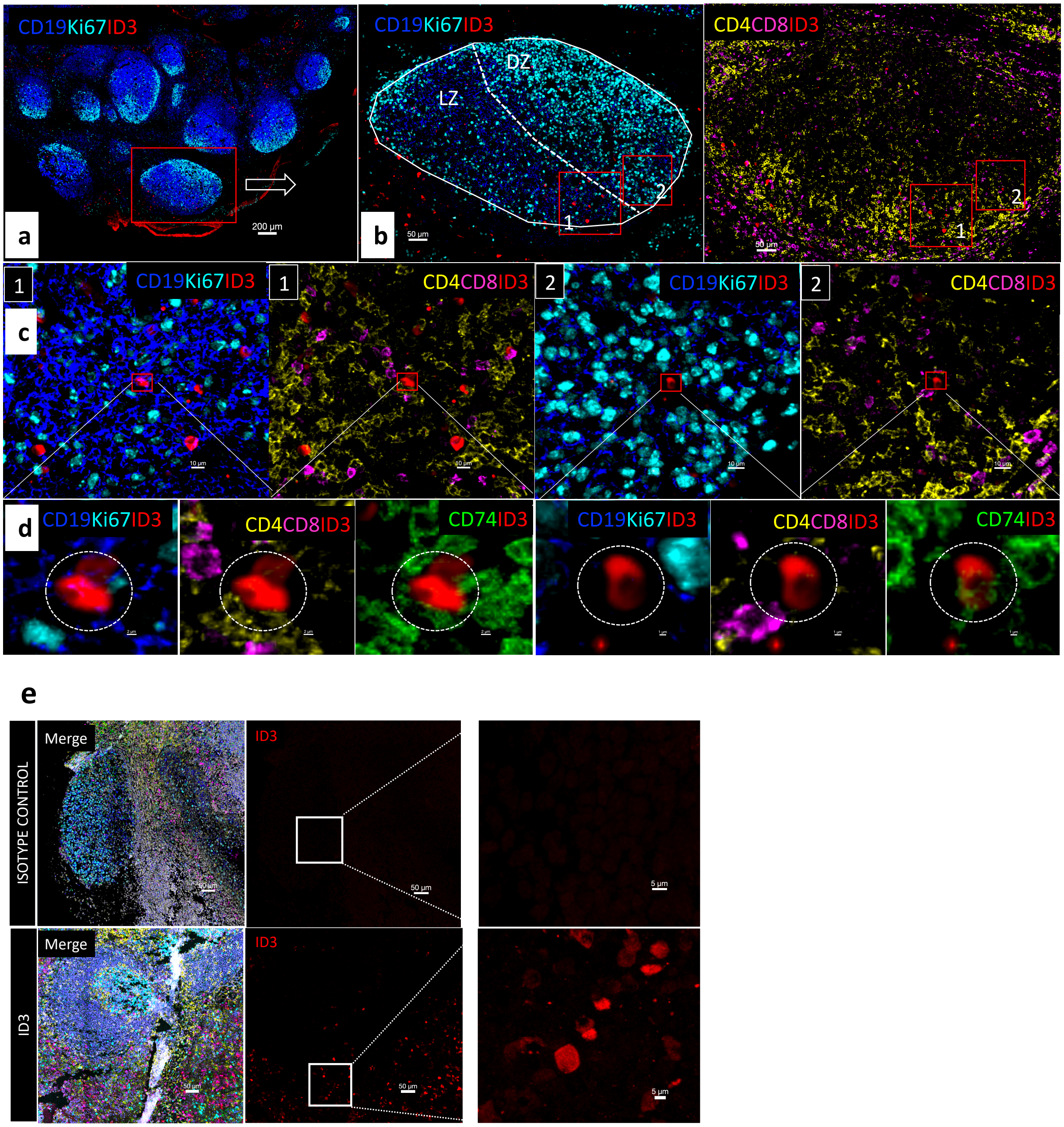

Supplementary Figure 4. Localization of ILC$_{FR}$ in tonsillar B cell follicles and isotype controls. (a) Confocal images showing the tonsil area imaged, distribution of B cell follicles as denoted by CD19 (blue) and Ki67 as well as ID3 positive cells (red). (b) Close up of a B cell follicle. Dotted lines demarcate the area of the follicle (LZ) as well as the dark zone (DZ) as

defined by the density of Ki67 staining (cyan). B cells are shown in blue (CD19), proliferating cells in cyan ($Ki67^+$), ID3 in red, CD4 in yellow and CD8 in magenta. (c) Zoomed in details of the red rectangular enclosures shown in (b). The location of a $CD74^+$ (green) $ID3^+$ (red) cell is shown with respect to the positioning of $CD19^+$ (blue) Ki67 (cyan) cells or $CD8^+$ (magenta) and $CD4^+$ (yellow) lymphoid cells. (d) Zoomed in close-ups confirming the positioning and phenotype of ILC_{FR} (CD19-CD4-CD8-CD74+ID3+). Images were acquired at 40x (NA 1.3) with no zoom. Images shown are sequential digital magnifications of 200um (a), 50um (b) 10um (c) and 1-2um (d) (lower panel close-ups). (e) Confocal images showing the specificity of the ID3 staining (red) and its isotype control (top row). Images shown are sequential digital magnifications of 50um and 5um (close-ups). A total of five biologically independent human tonsils were imaged.

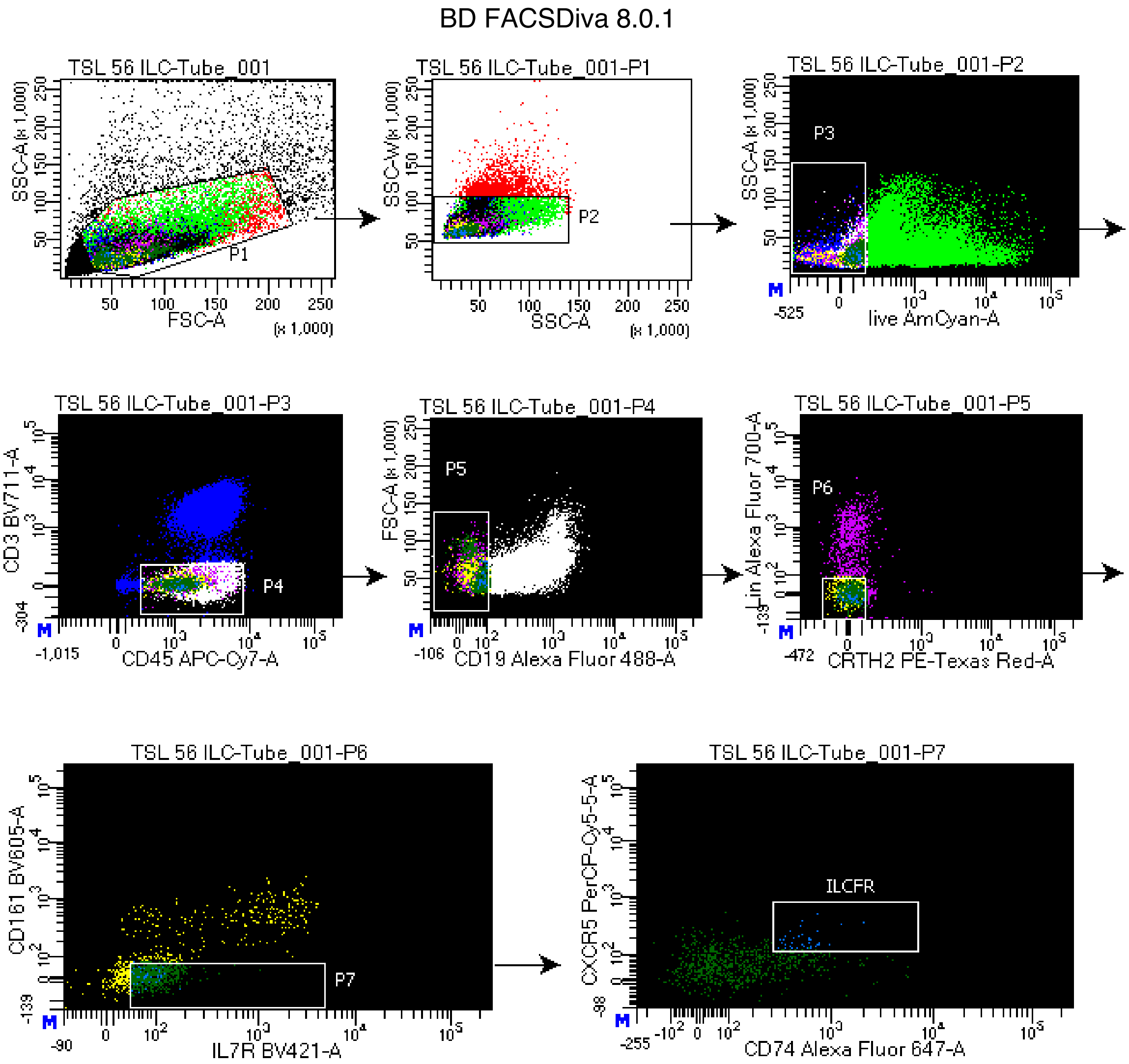

Supplementary Figure 5. Sort gating strategy to stringently identify ILC_{FR}

Representative conventional flow cytometry plots from an adult tonsil showing the more stringent and hierarchical sorting gating strategy from singlet TMNC lymphocytes (FSA vs. SSA and SSW vs. SSA), to live cells (AmCyan negative P3), to CD45$^+$ white blood cells that are not T cells (CD3 negative P4), to non B cells (CD19 negative P5), to lineage negative (Lin= CD4, CD11b, CD11c, CD14, CD16), and CRTH2 negative (P6), to CD161 negative and IL7R intermediate (P7), to finally CXCR5$^+$ and C74$^+$ ILC subset (ILC_{FR}) (blue) populations indicated by arrows and numbered gates.

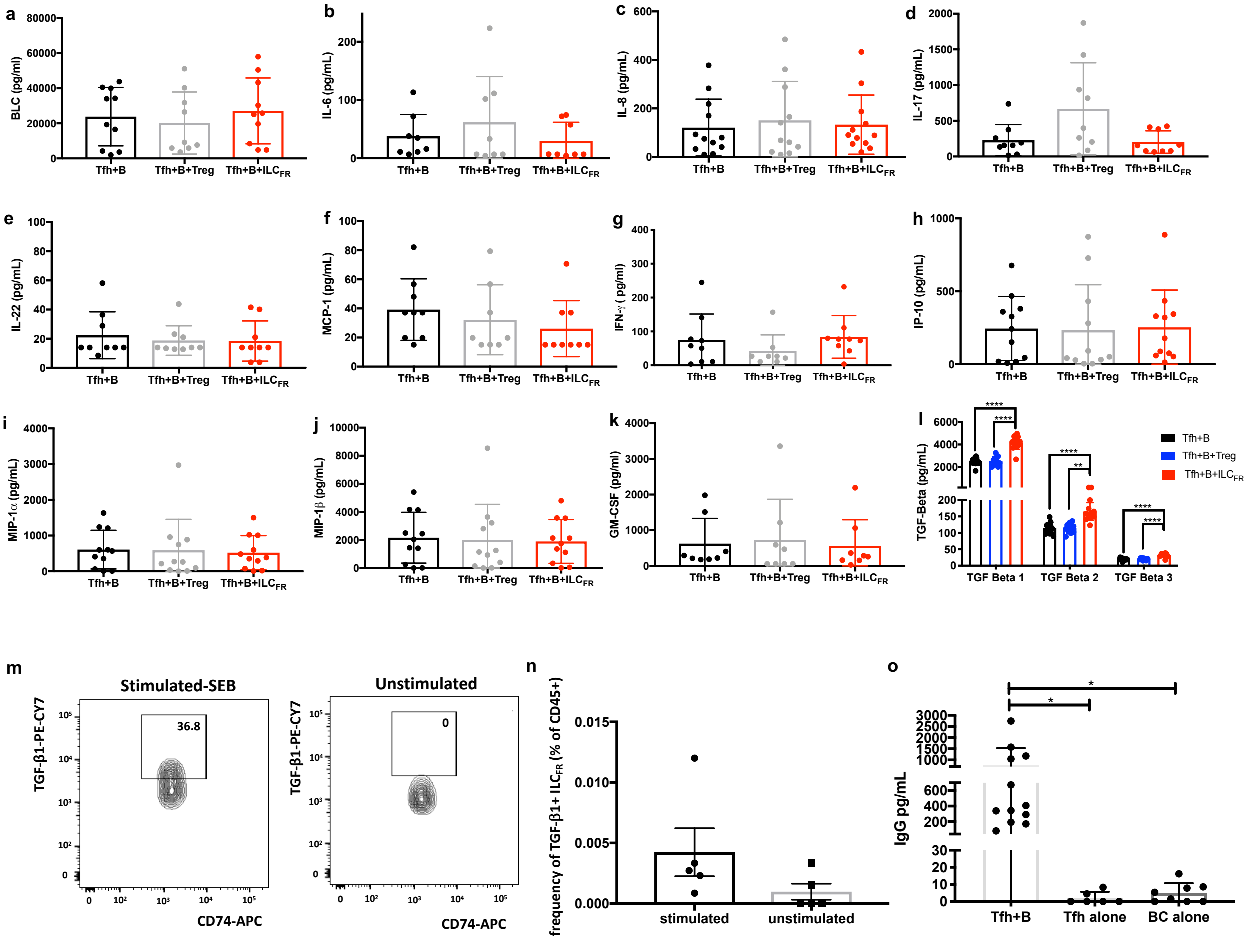

Supplementary Figure 6. Cytokine production from ILC$_{FR}$ and co-culture of GC-Tfh and GC-B cells with Treg or ILC$_{FR}$ plus stimulation controls

The graphs shows production of (a) BLC, (b) IL-6, (c) IL-8, (d) IL-17, (e) IL-22, (f) MCP-1, (g) INF-γ, (h) IP-10, (i) MIP-1α, (j) MIP-1β, (k) GM-CSF, and (l) TGF-β in the supernatants of 5-day co-culture of 30,000 GC-Tfh with 30,000 autologous GC-B cells (black) plus or minus addition of 15,000 Tregs (grey) or 1,000 ILC$_{FR}$ (red) in the presence of 100ng/mL SEB superantigen measured by Luminex. (n = 10 or 12 biologically independent tonsils per group). In (m) we see

TGF-β production from ILC_{FR} after 24h stimulation with 1ug/ml SEB quantified in (n) for 5 biologically independent tonsils (not significant). (o) shows production of IgG in the supernatants of 5-day co-culture of 30,000 GC-Tfh with 30,000 autologous GC-B cells or 30,000 GC-B alone, or 30,000 GC-Tfh alone all in the presence of 100ng/mL SEB superantigen measured by ELISA (n = 10 or 12 biologically individual tonsils per group, 4 independent experiments). Tfh+B vs. Tfh alone: (Paired, two-tailed parametric t-test; *p = 0.034; t= 3.163, df = 4, 95% CI (-1185 to -77.08), Mean ± SD), and for Tfh+B vs. B cells alone: (Paired, two-tailed parametric t-test; *p = 0.017; t= 3.258, df = 6, 95% CI (-869.7 to 127.5), Mean ± SD). For TGF-β co-culture analysis in (l): (Paired, two-tailed parametric t-test; β1: Tfh+B vs. ILC_{FR}: ****p < 0.0001, df = 11, 95% CI: (54.79 to 83.75), t = 10.53, Tfh+B vs. Treg: ****p < 0.0001, df = 10, 95% CI: (47.33 to 84.51), t = 7.90, for β2: Tfh+B vs. ILC_{FR}: ***p = 0.0001, df = 11, 95% CI: (28.9 to 65.55), t = 5.67, Tfh+B vs. Treg: ***p = .001, df = 10, 95% CI: (20.69 to 59.26), t = 4.62, β3: Tfh+B vs. ILC_{FR}: ****p < 0.0001, df = 11, 95% CI: (52.03 to 81.31), t = 10.02, Tfh+B vs. Treg: ****p < 0.0001, df = 10, 95% CI: (42.47 to 78.5), t = 7.48; Mean ± SD) (4 independent experiments).

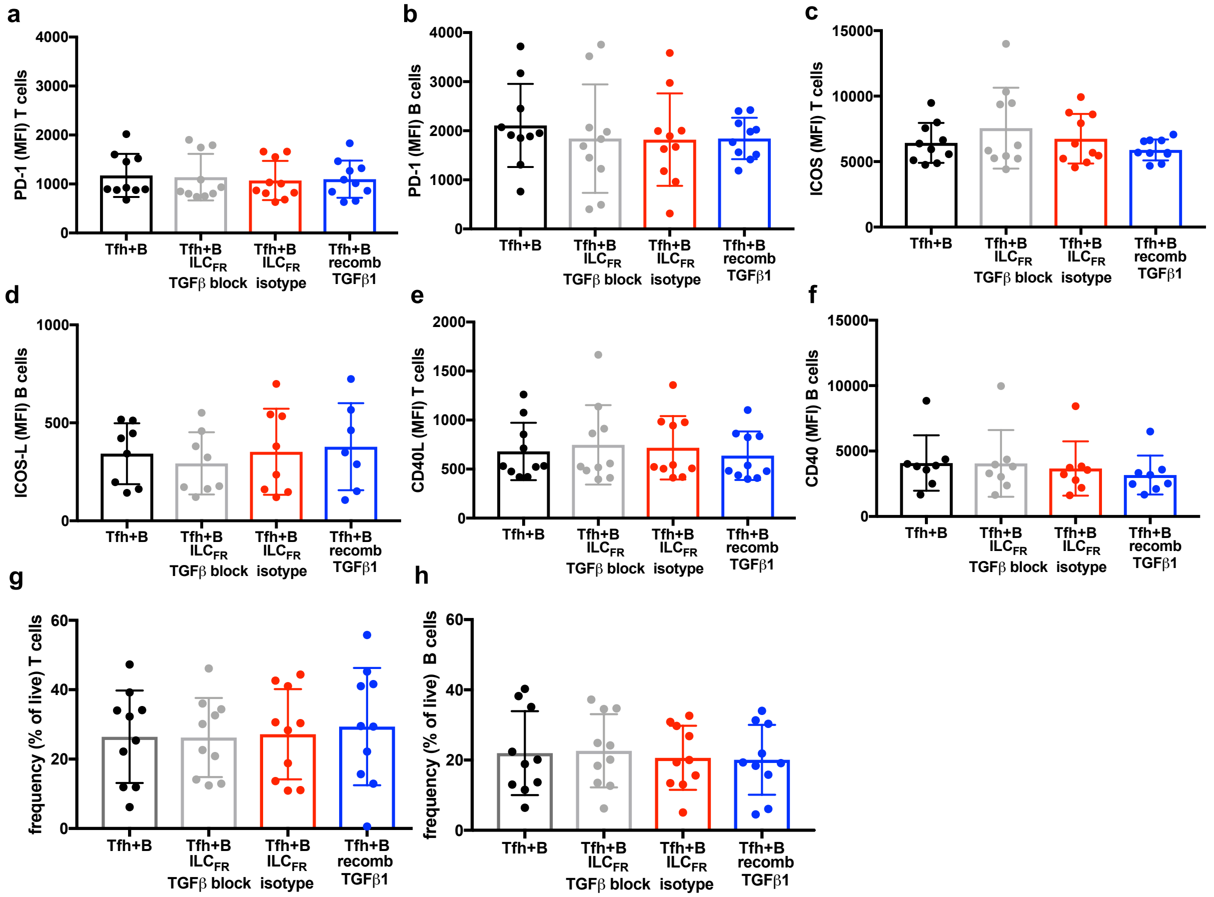

Supplementary Fig. 7. Co-stimulatory markers and viability for GC-Tfh or GC-B cells after co-cultures

Graphs represent frequency of GC-Tfh or GC-B subsets ex vivo after 5 days in co-culture with 30,000 GC-Tfh and 30,000 GC-B (black) plus or minus 1,000 ILC_{FR} with or without TGF-β blocking (grey) or isotype control (red), plus or minus rhTGF-β1 protein (blue) in the presence of 100ng/mL SEB superantigen analyzed by flow cytometry. Graphs show PD-1 expression on (a) GC-Tfh, (b) GC-B, (c) ICOS expression on GC-Tfh, (d) ICOS-L expression on GC-B, (e) CD40 expression on GC-B, and (f) CD40-L expression on GC-Tfh. In (g) the graph represents frequency of live (Vivid dead staining negative) GC-Tfh cells or (h) GC-B cells after the 5-day co-culture. (n = 10

biologically independent tonsils per group, 3-4 independent experiments, bars represent Mean ± SD).

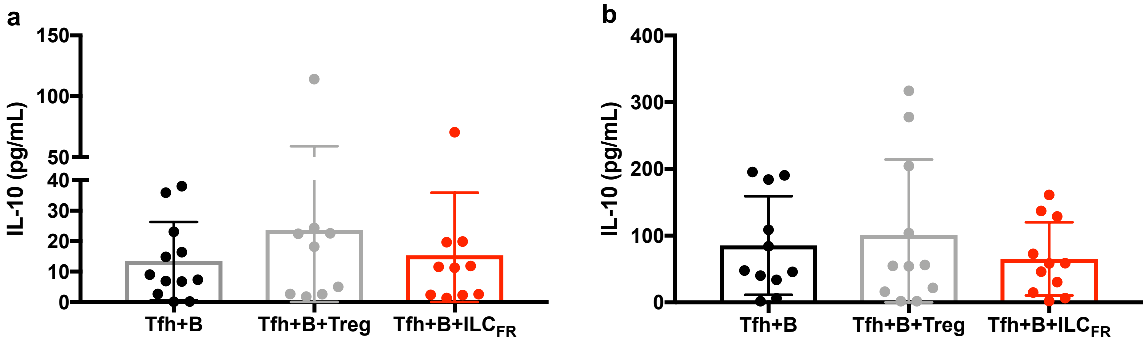

Supplementary Fig. 8. IL-10 levels are unchanged in the presence of ILC_{FR}

(a) The graph represents IL-10 production in the supernatants of 5-day co-culture of 30,000 GC-Tfh with 30,000 autologous GC-B cells (black) plus or minus addition of 15,000 Tregs (grey) or 1,000 ILC_{FR} (red) in the presence of 100ng/mL SEB superantigen measured by (a) ELISA or (b) Luminex. (n = 9 or 12 biologically independent tonsils per group, 3-4 independent experiments) (bars represent Mean ± SD).
