# Supplementary material for: A follicular regulatory Innate Lymphoid Cell population impairs interactions between germinal center Tfh and B cells
Source: Commun Biol. 2021 May 12;4:563. doi: 10.1038/s42003-021-02079-0 (PMC8115650; doi:10.1038/s42003-021-02079-0)
Supplement: Supplementary file 2 — Reporting Summary [file 42003_2021_2079_MOESM2_ESM.pdf]

nature research

Corresponding author(s): Elias Haddad
Virginie Tardif

Last updated by author(s): Mar 8, 2021

Reporting Summary

Nature Research wishes to improve the reproducibility of the work that we publish. This form provides structure for consistency and transparency in reporting. For further information on Nature Research policies, seeAuthors & Referees and theEditorial Policy Checklist .

Statistics

For all statistical analyses, confirm that the following items are present in the figure legend, table legend, main text, or Methods section.

n/a	Confirmed	
☐	☒	The exact sample size (*n*) for each experimental group/condition, given as a discrete number and unit of measurement
☐	☒	A statement on whether measurements were taken from distinct samples or whether the same sample was measured repeatedly
☐	☒	The statistical test(s) used AND whether they are one- or two-sided<br>*Only common tests should be described solely by name; describe more complex techniques in the Methods section.*
☐	☒	A description of all covariates tested
☐	☒	A description of any assumptions or corrections, such as tests of normality and adjustment for multiple comparisons
☐	☒	A full description of the statistical parameters including central tendency (e.g. means) or other basic estimates (e.g. regression coefficient) AND variation (e.g. standard deviation) or associated estimates of uncertainty (e.g. confidence intervals)
☐	☒	For null hypothesis testing, the test statistic (e.g. *F*, *t*, *r*) with confidence intervals, effect sizes, degrees of freedom and *P* value noted<br>*Give P values as exact values whenever suitable.*
☒	☐	For Bayesian analysis, information on the choice of priors and Markov chain Monte Carlo settings
☒	☐	For hierarchical and complex designs, identification of the appropriate level for tests and full reporting of outcomes
☒	☐	Estimates of effect sizes (e.g. Cohen's *d*, Pearson's *r*), indicating how they were calculated

Our web collection on statistics for biologists contains articles on many of the points above.

Software and code

Policy information about availability of computer code

Data collection	n/a
Data analysis	n/a

For manuscripts utilizing custom algorithms or software that are central to the research but not yet described in published literature, software must be made available to editors/reviewers. We strongly encourage code deposition in a community repository (e.g. GitHub). See the Nature Research guidelines for submitting code & software for further information.

Data

Policy information about availability of data

All manuscripts must include a data availability statement. This statement should provide the following information, where applicable:
- Accession codes, unique identifiers, or web links for publicly available datasets
- A list of figures that have associated raw data
- A description of any restrictions on data availability

Sequence and gene expression data are available at the Gene Expression Omnibus (accession number pending).

Field-specific reporting

Please select the one below that is the best fit for your research. If you are not sure, read the appropriate sections before making your selection.

☒ Life sciences ☐ Behavioural & social sciences ☐ Ecological, evolutionary & environmental sciences

For a reference copy of the document with all sections, see nature.com/documents/nr-reporting-summary-flat.pdf

Life sciences study design

All studies must disclose on these points even when the disclosure is negative.

Sample size	Sample sizes were not predetermined based on statistical methods, but were chosen according to the standards of the field (at least four independent human tonsil samples were used for each condition, most times 7-10 were used). This is due to previous work and takes into account the heterogeneity of human samples.
Data exclusions	no data was excluded from consideration.
Replication	Reported results were consistently replicated across multiple experiments with all replicates generating similar results. There were at least 2 replicates for each experiment, usually 4-6 replicates.
Randomization	Each human tonsil was used for all experimental subgroups so there were both autologous and heterologous controls for each human subject.
Blinding	When performing ELISA and Luminex experiments on co-culture supernatants, samples were blinded and given a number to keep from bias.

Reporting for specific materials, systems and methods

We require information from authors about some types of materials, experimental systems and methods used in many studies. Here, indicate whether each material, system or method listed is relevant to your study. If you are not sure if a list item applies to your research, read the appropriate section before selecting a response.

Materials & experimental systems

n/a	Involved in the study
☐	☒ Antibodies
☒	☐ Eukaryotic cell lines
☒	☐ Palaeontology
☒	☐ Animals and other organisms
☐	☒ Human research participants
☐	☒ Clinical data

Methods

n/a	Involved in the study
☒	☐ ChIP-seq
☐	☒ Flow cytometry
☒	☐ MRI-based neuroimaging

Antibodies

Antibodies used	All antibodies are listed in supplementary information with supplier name, clone number and catalog number
Validation	antibodies were used as per manufacturers recommendation for concentration and titrated as necessary

Human research participants

Policy information about studies involving human research participants

Population characteristics	Tonsils were obtained from patients age 17-50 with a mix of both males and females. Samples used were from patients who were having their tonsils removed due to chronic infections.
Recruitment	Participants were recruited who were having their tonsils taken removed due to repetitive chronic infections of the area
Ethics oversight	Martin Memorial Health Systems (Florida), St. Christopher's Hospital for Children (Pennsylvania) and National Institutes of Health Clinical Research Center (Bethesda). The Institutional Review Boards at the relevant institutions approved all procedures, and all participants provided signed informed consent.

Note that full information on the approval of the study protocol must also be provided in the manuscript.

Clinical data

Policy information about clinical studies

All manuscripts should comply with the ICMJEguidelines for publication of clinical research and a completedCONSORT checklist must be included with all submissions.

Clinical trial registration	ClinicalTrials.gov Identifier: NCT00001316
Study protocol	under the link above and using the identifier listed, one can access the full study protocol
Data collection	LN mononuclear cells collected from HIV+ patients for use in our study

Outcomes	n/a

Flow Cytometry

Plots

Confirm that:

- [x] The axis labels state the marker and fluorochrome used (e.g. CD4-FITC).
- [x] The axis scales are clearly visible. Include numbers along axes only for bottom left plot of group (a 'group' is an analysis of identical markers).
- [x] All plots are contour plots with outliers or pseudocolor plots.
- [x] A numerical value for number of cells or percentage (with statistics) is provided.

Methodology

Sample preparation	Sample preparation listed in Methods
Instrument	FACS Aria Fusion, and Fortessa
Software	FACSDiva for collection and data analyzed using FlowJo (v10)
Cell population abundance	100-200 million TMNC were sorted per patient to acquire relevant cell populations: 300,000 GCB and GCTfh, 1,000 ILCFR, 15,000 Treg, 3,000 ILC3<br>Purity was assessed by staining for cell specific markers and validated by RNAseq data
Gating strategy	Relevant gating strategies shown in Figure 1 and the Supplementary Information

- [x] Tick this box to confirm that a figure exemplifying the gating strategy is provided in the Supplementary Information.
